# Endophenotype Research in Epilepsy Across Time

**DOI:** 10.3390/brainsci15121275

**Published:** 2025-11-27

**Authors:** Ozgun Yetkin, Ovinuchi Ejiohuo, Betul Baykan, Marcin Zarowski

**Affiliations:** 1 Department of Developmental Neurology, Poznan University of Medical Sciences, 60-355 Poznan, Poland; zarowski@ump.edu.pl; 2 Doctoral School, Poznan University of Medical Sciences, 60-812 Poznan, Poland; ovinuchi.ejiohuo@faculty.umgc.edu; 3 Department of Biology, University of Maryland Global Campus, UMGC in Europe, 60-554 Poznan, Poland; 4 EMAR Medical Center, Department of Neurology, 34367 Istanbul, Türkiye

**Keywords:** epilepsy, endophenotypes, bibliometric analysis, transdiagnostic markers

## Abstract

**Background/Objectives**: Endophenotypes—quantifiable biological markers bridging genetic variations and clinical manifestations—have significantly evolved since their introduction to psychiatric genetics. This study presents the first comprehensive analysis of endophenotype research in epilepsy, examining validation frameworks, methodological approaches, and the potential for clinical translation. **Methods**: We employed a dual-methodological approach combining the bibliometric analysis with a systematic review and a meta-analysis. Literature searches in the Web of Science and Scopus databases (17 July 2025) employed comprehensive strategies that incorporated endophenotype and epilepsy terminology. In the bibliometric analysis, the ‘Bibliometrix’ R package (version 4.4.3 (R Core Team, 2024) was used for publication trends, collaboration networks, and thematic evolution. The meta-analysis quantitatively synthesized validation outcomes across studies. For the systematic review, we compared traditional validation criteria with the Endophenotype 2.0 framework and applied machine learning-based validation techniques across 53 studies meeting rigorous inclusion criteria. **Results**: An analysis of 169 publications (2001–2025) revealed moderate annual growth (6.94%) with acceleration after 2015. Neuroimaging features achieved exceptional validation rates (77.8% perfect scores under Endophenotype 2.0), with functional MRI studies reaching 87.5% success. The Endophenotype 2.0 framework significantly outperformed traditional criteria (58.5% vs. 43.4%), particularly for genetic/molecular endophenotypes (83.3% vs. 0%). Family-based designs emerged as the strongest validation predictors (96% vs. 25% for population-based studies). International collaboration remained limited (4.1%). **Conclusions**: The endophenotype research in epilepsy has evolved toward validated biomarkers. The more comprehensive performance of the novel validation framework positions multiple endophenotypes—particularly neuroimaging and genetic markers—for the implementation of precision medicine. Our findings reveal opportunities for transdiagnostic biomarkers that could revolutionize risk assessment, early intervention, and personalized treatment across neurodevelopmental conditions.

## 1. Introduction

Epilepsy affects over 50 million people worldwide, representing a significant burden on patients, families, and healthcare systems [1]. However, despite decades of rigorous research and the development of advanced genomic technology, it remains unclear how genetic variants relate to seizure susceptibility [2]. What clinicians refer to as epilepsy actually includes a diverse range of disorders with a variety of etiologies, from complex polygenic architectures underlying common focal and generalized epilepsies to single-gene mutations causing rare epileptic encephalopathies [3].

Endophenotypes are measurable, heritable components that exist midway between genes and complex clinical disorders, thereby bridging the genotype and phenotype. They were first described by Gottesman and Gould in their work on the genetics of schizophrenia [4]. Endophenotypes offer a more direct biological link to disease etiology than broad clinical phenotypes, as they capture heritable characteristics that consistently reflect dysfunction in specific biological systems [4,5]. The field has been recently revolutionized by Liu and Gershon’s Endophenotype 2.0 framework, published in 2024, which redefines endophenotypes as genetically influenced phenotypes linked to disease or treatment characteristics and their related events [6]. 

Distinct concepts include endophenotypes, biomarkers, and surrogate endpoints. Endophenotypes are heritable traits linking genetic variation and clinical disease, characterized by familial aggregation and genetic mediation [4]. Biomarkers are biological indicators reflecting normal or pathological processes, not requiring genetic involvement. A specific subset, surrogate endpoints, are validated indicators in trials that predict clinical outcomes [7]. While all endophenotypes are biomarkers, not all biomarkers qualify as endophenotypes; only those with causal links to outcomes can be surrogate endpoints. Endophenotypes provide a balance between mechanistic insight and clinical utility, indicating rigorous validation standards [8,9].

Endophenotypes are increasingly recognized as transdiagnostic markers that transcend traditional diagnostic boundaries, offering insights into shared neurobiological mechanisms across psychiatric and neurological conditions. Recent work in developmental psychopathology has demonstrated that cortical morphological alterations—particularly reduced surface area and altered thickness—predict dimensional psychopathology (internalizing and externalizing profiles) across childhood and adolescence, independent of categorical diagnoses [10,11]. These cortical phenotypes show stronger associations with general psychopathology factors than with disorder-specific categories, suggesting that underlying neurodevelopmental deviations contribute to comorbidity patterns [11,12].

Epilepsy shares substantial genetic and neurobiological overlap with other neurodevelopmental disorders, including autism spectrum disorder, intellectual disability, and attention-deficit/hyperactivity disorder, with common pathways involving excitatory/inhibitory balance, synaptic signaling, and ion channel function [13,14]. This positions epilepsy endophenotypes within a broader transdiagnostic framework, where markers such as executive dysfunction, altered prefrontal-limbic connectivity, and cortical structural abnormalities may reflect shared vulnerabilities across multiple neurodevelopmental and psychiatric conditions [15].

Due to its phenotypic heterogeneity and complex inheritance patterns, epilepsy presents unique challenges for genetic research [16]. Unlike monogenic disorders, epilepsies encompass diverse seizure types, syndromes, and etiologies, each potentially involving distinct genetic architectures [17]. Endophenotypes offer a particularly compelling solution to these challenges by providing measurable, biologically informed traits closer to gene action than clinical syndromes. In epilepsy research, endophenotypes can identify specific neurophysiological abnormalities, structural brain changes detectable by neuroimaging, or cognitive patterns that may be shared among unaffected family members [18]. Beyond helping identify epilepsy genes, this approach offers insights into the disease mechanisms that may guide the development of anti-epileptogenic drugs rather than just suppressing seizure symptoms [19]. While endophenotypes provide valuable insights, they remain an empirical concept in epilepsy research. A major challenge is their limited specificity, as many proposed endophenotypes are shared across epilepsy syndromes and even other neurodevelopmental disorders, complicating precise syndrome differentiation [20].

However, significant methodological challenges and scoring inconsistencies exist when using endophenotype validation criteria in epilepsy research. The shift from conventional five-criterion models (association with illness, heritability, state independence, familial cosegregation, and higher frequency in unaffected relatives) to the more straightforward Endophenotype 2.0 framework (reliable measurement, association with disease/treatment, and genetic mediation) addresses previous validation difficulties [6].

Despite increasing interest in endophenotype-based approaches, no comprehensive analysis has been conducted to examine how these concepts have been explicitly applied to epilepsy research. Our study aims to map this landscape through complementary lenses: a bibliometric analysis to quantify publication trends, collaboration patterns, and thematic evolution; and a systematic review to assess methodological quality, validation rigor, and potential clinical translation [21].

Four key questions guided our investigation: (1) How has epilepsy endophenotype research evolved? (2) Which epilepsy syndromes have received the most attention? (3) How well do existing studies satisfy different validation frameworks? (4) What are the commonly investigated endophenotypes, and their precision medicine potential?

## 2. Materials and Methods

### 2.1. Study Design and Search Strategy

This study combined a bibliometric analysis with a systematic review to explore scientific trends and validate epilepsy endophenotypes.

Our search strategy is designed to capture the full spectrum of endophenotype-related research in epilepsy. We selected the Web of Science Core Collection (WoSCC) as our primary database, given its comprehensive citation tracking capabilities and standardized bibliographic data formatting, both of which are essential for a network analysis. Scopus was employed as a secondary database to provide broader coverage. The search strategy was developed using a comprehensive combination of Medical Subject Headings (MeSH) terms and free-text keywords related to endophenotypes and epilepsy. The literature search was conducted without a temporal limit. The entire search strategy, including Boolean operators, is detailed in Table 1 and was conducted on 17 July 2025.

### 2.2. Data Extraction and Processing

We exported complete bibliographic records (author information, institutional affiliations, publication years, journal sources, abstracts, keywords, and citation data) in formats optimized for bibliometric analysis (Excel and CSV). Initial deduplications were checked automatically using R algorithms, and then, residual duplications were manually eliminated.

Two reviewers (O.Y. and O.E.) independently evaluated titles, abstracts, and full texts using the Rayyan systematic review platform, facilitating blind screening and conflict resolution [22]. Disagreements were resolved through discussion and, when necessary, consultation with senior authors. Data extraction was performed using a standardized extraction form that covered the following aspects: study characteristics (author, year, country, and study design), population details (sample size, epilepsy type, and demographics), endophenotype methodology (measurement tools and validation criteria), key findings, and quality assessment parameters. The R statistical software, version 4.4.3 (R Core Team, 2024) [23], was used. The ‘bibliometrix’ package (version 4.3.0), accessed via the Biblioshiny web interface, was used for the bibliometric analysis [23,24].

This research was registered on the Open Science Framework (OSF) to promote transparency and accountability in our research methodology. The full registration, including the detailed study protocol, analysis plan, and data management procedures, is publicly available at https://doi.org/10.17605/OSF.IO/QBY4F.

### 2.3. Study Selection Process

A two-phase screening process was implemented to address the study’s dual objectives.

Phase 1. We applied intentionally broad inclusion criteria to establish the overall research landscape, with any publication types (original research, reviews, case studies, and conference proceedings) addressing endophenotypes or intermediate phenotypes in the context of epilepsy research, conducted both human and animal subjects, and had no restrictions on publication year, language, or study design, with exclusion criteria of articles not mentioning epilepsy or seizure disorders, and no relevance to endophenotype concepts.

**Figure 1:**
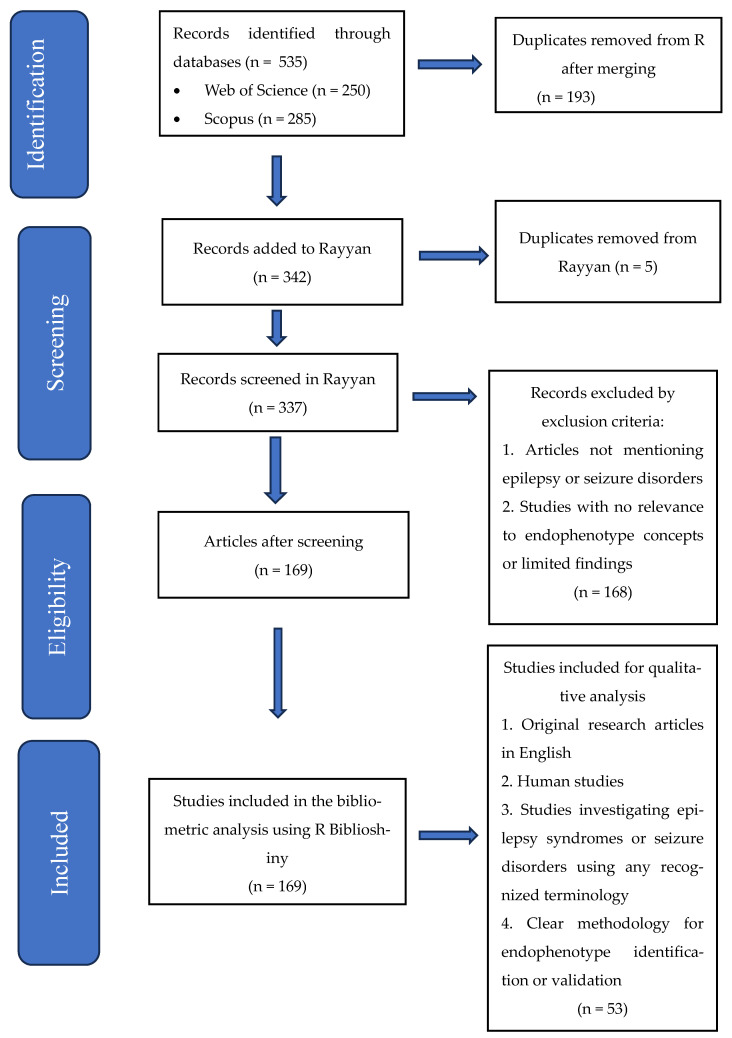
Preferred Reporting Items for Systematic reviews and Meta-Analyses (PRISMA) flow diagram of the study methodology.

Phase 2. We implemented more stringent criteria for the systematic review component. We restricted our analysis to original research articles published in English that studied endophenotypes in human epilepsy populations. Conference abstracts, review articles without original data, and purely theoretical papers were excluded at this stage. Figure 1 shows the PRISMA flowchart of the study methodology.

### 2.4. Quality Assessment, Risk of Bias, and Certainty of Evidence

The methodological quality and certainty of evidence were evaluated using two complementary approaches: individual study quality assessment and evidence certainty by endophenotype class.

The methodological quality of all included observational studies was assessed using the Newcastle-Ottawa Scale (NOS), a validated tool for evaluating non-randomized studies in systematic reviews [25]. The NOS evaluates studies across three domains: selection of study groups (maximum of four stars), comparability of groups (maximum two stars), and ascertainment of exposure/outcome (maximum of three stars), with a maximum total score of nine stars. Studies were classified based on their total NOS scores as follows: high quality (seven to nine stars), medium quality (four to six stars), or low quality (zero to three stars). Two independent reviewers (O.Y. and O.E.) conducted quality assessments for all included studies, with discrepancies resolved through discussion to reach a consensus. Detailed NOS ratings for each study are provided in Supplementary Table S4.

We applied GRADE (Grading of Recommendations Assessment, Development and Evaluation) principles to assess the overall certainty of evidence for each endophenotype class (neuroimaging, electrophysiological, cognitive, genetic/molecular, psychiatric, and clinical phenotyping). Although GRADE was originally developed for intervention studies, we adapted its framework to evaluate observational endophenotype validation research. Evidence certainty was rated as high, moderate-high, moderate, or low based on consideration of study design quality (NOS scores), consistency of findings across studies, precision of validation rates, directness of evidence for endophenotype criteria, and risk of publication bias. For each endophenotype class, we synthesized the number of studies, validation success rate, key methodological strengths and limitations, and clinical readiness for implementation. GRADE-style certainty assessments are summarized in Supplementary Table S5.

### 2.5. Assessment of Validation Frameworks

A unique aspect of our investigation involved dual evaluation using traditional and modern endophenotype validation frameworks.

Traditional Framework (Gottesman and Gould, 2003) [4] scored studies on five criteria (from zero to five points):

1. Association with illness—a clear relationship between endophenotype and epilepsy diagnosis
2. Heritability—evidence from family/twin studies or formal heritability estimates
3. Family co-segregation—trait presence in patients’ family members
4. State-independence—trait persistence regardless of acute illness episodes or treatment
5. Higher frequency in unaffected relatives—intermediate trait expression compared to the general population

Endophenotype 2.0 Framework (Liu and Gershon, 2024) [6] assessed three criteria (from zero to three points):

1. Reliable measurement—validated, reproducible assessment methods
2. Association with disease/treatment—clear relationship with clinical outcomes or therapeutic response
3. Genetic mediation—evidence from molecular studies, family patterns, or heritability analysis

Two independent reviewers (O.Y. and O.E.) scored each study against both frameworks using standardized extraction forms (Supplementary Table S1). For each criterion, studies received binary scores (met/not met), with percentage compliance calculated for comparative analysis. Discrepancies were discussed to reach a consensus. Complete operational definitions, including binary scoring criteria, adjudication rules for ambiguous cases, and example-based coding scenarios drawn from the included studies, are provided in Supplementary Table S2. The codebook includes specific examples from the systematic review that demonstrate typical evidence satisfying or failing to satisfy each criterion, thereby enhancing reproducibility and enabling consistent application of validation standards.

To quantify inter-rater reliability, Cohen’s Kappa (κ) statistics were computed using unweighted (nominal), linear-weighted, and quadratic-weighted models to capture increasing sensitivity to the ordinal nature of the scoring system [26,27]. Unweighted Kappa assessed exact categorical agreement, while linear and quadratic weighting accounted for the ordered structure of the ratings by penalizing larger disagreements more heavily. Agreement strength was interpreted using established guidelines [26,28].

For each endophenotype phase, ratings from both raters were summarized in a confusion matrix, from which the total number of exact matches and the simple percent agreement were calculated. Quadratic-weighted Kappa was selected as the primary reliability index, given its suitability for ordinal scales and its superior ability to reflect clinically meaningful gradations in scoring differences. All analyses were conducted in R (version 4.2.1) using the ‘irr’ package (Supplementary Table S3).

### 2.6. Meta-Analysis of Validation Proportions and Meta-Regression

A random-effects logistic meta-analysis was conducted on the log-odds of achieving a perfect score. Data were extracted from the 53 studies, including sample size, design, modality, and publication year. For each study, the proportion achieving a perfect score was converted to log-odds, with a continuity correction of 0.5 applied to zero cells. Sampling variances were computed using standard formulas for log-odds, and pooled estimates were obtained using restricted maximum likelihood (REML). Heterogeneity was quantified using τ^2^, I^2^, and the Q statistic.

To explore sources of heterogeneity, we performed meta-regression with the following moderators: publication year, log-transformed sample size, study design (family-based vs. population-based), and modality. Univariable meta-regressions were first conducted for each moderator, followed by a multivariable model including all predictors. Effect estimates were reported as log odds and transformed into odds ratios (ORs) with 95% confidence intervals. All analyses were performed using the ‘metafor’ and ‘meta’ packages in R.

### 2.7. Sensitivity Analysis

The robustness of the meta-analytic results was evaluated using the leave-one-out analysis and multicomponent influence diagnostics. For the leave-one-out procedure, each study was sequentially removed, and the pooled effect size (τ^2^) and Q statistic were recalculated to assess the model’s stability. Influence diagnostics were conducted using externally standardized residuals (rstudent), Difference in Fits (DFFITS), Cook’s distance (cook.d), covariance ratios cov.r), τ^2^-deletion (Tau-squared Delete), and Q-deletion statistics (QE.del) to identify outliers and influential studies. Leverage (hat) and study weights were examined to assess the contribution of each study to model precision and overall influence. All diagnostics were implemented within the random-effects framework, using predefined thresholds to flag potentially influential observations (e.g., residuals ±2, Cook’s distance > 4/*n*). Together, these analyses tested whether the pooled effect was sensitive to individual studies and whether any single study disproportionately contributed to heterogeneity.

### 2.8. Machine Learning Prediction of Validation Score Using a Random Forest Model

To further validate the predictors of the validation score, we create a random forest machine learning model for Endophenotype 2.0. Validation scores were operationalized as a binary outcome with studies achieving perfect validation scores (three) vs. those with lower scores (one or two). This binary classification approach was chosen to optimize model interpretability and address the class imbalance in the ordinal score distribution. Our variables study design, modality, sample size, and publication year were chosen as the predictor variables. The data were randomly partitioned into training (70%, n = 37) and testing (30%, n = 16) sets using stratified sampling to maintain the distribution of outcomes across the sets [29]. A random seed (123) was set to ensure reproducibility.

Random forest models were implemented using the ‘randomForest’ package in R. Two modeling approaches were evaluated. First, the multiclass classification predicts exact scores (1, 2, or 3), and then the binary classification predicts perfect score achievement (our primary approach) [30]. Variable importance was calculated using both mean decrease in accuracy (permutation importance) and mean decrease in Gini impurity [31,32]. The model performance was assessed using accuracy, sensitivity (true positive rate), specificity (true negative rate), positive predictive value (precision), and FI score. Out-of-bag (OOB) error rates from the training process provided internal validation estimates [33]. Receiver operating characteristic (ROC) curves were generated for the test set, and the area under the curve (AUC) with a 95% confidence interval was quantified to assess discriminatory ability. The optimal probability threshold was determined using Youden’s Index [34]. Training and test set AUC values were compared to evaluate potential overfitting, with differences < 0.10 considered acceptable [35,36].

## 3. Results

### 3.1. Bibliometric Analysis Results

**Figure 2:**
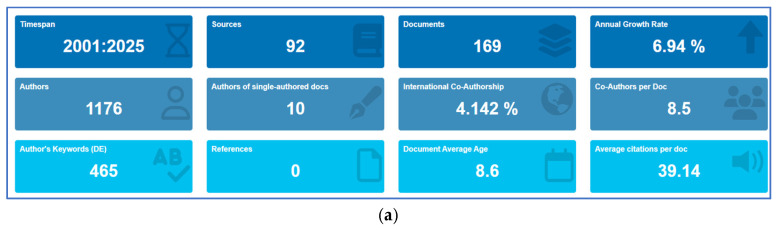

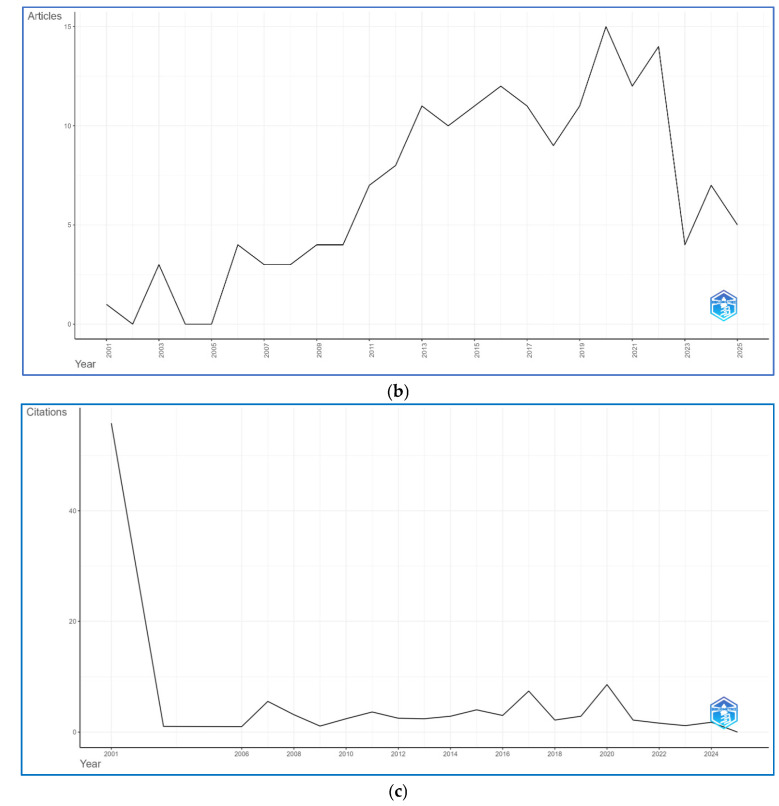
Results of the bibliometric analysis showing (**a**) descriptive summary, (**b**) annual scientific production, and (**c**) annual citation over time.

Bibliometric analysis identified 169 publications spanning the years 2001–2025, sourced from 92 sources. The field showed moderate growth (6.94% annually) with 1176 authors averaging 8.5 collaborators per document. Publications averaged 39.14 citations, with a mean time since publication of 8.6 years. However, international co-authorship remained low at 4.1% (Figure 2a).

Scientific output underwent three distinct phases, emerging (2001–2005, minimal activity), growth (2006–2010, gradual increase), and expansion (peaked 2019–2020, declining from 2021) (Figure 2b).

An article citation analysis from 2001 to 2024 reveals a striking pattern of citations, starting with an exceptionally high peak of more than 50 citations in 2001 and then declining sharply to almost zero by 2005–2006 and remaining steady at a much lower baseline of roughly two to eight citations annually with slight fluctuations in 2007, 2017, and 2020 (Figure 2c).

**Figure 3:**
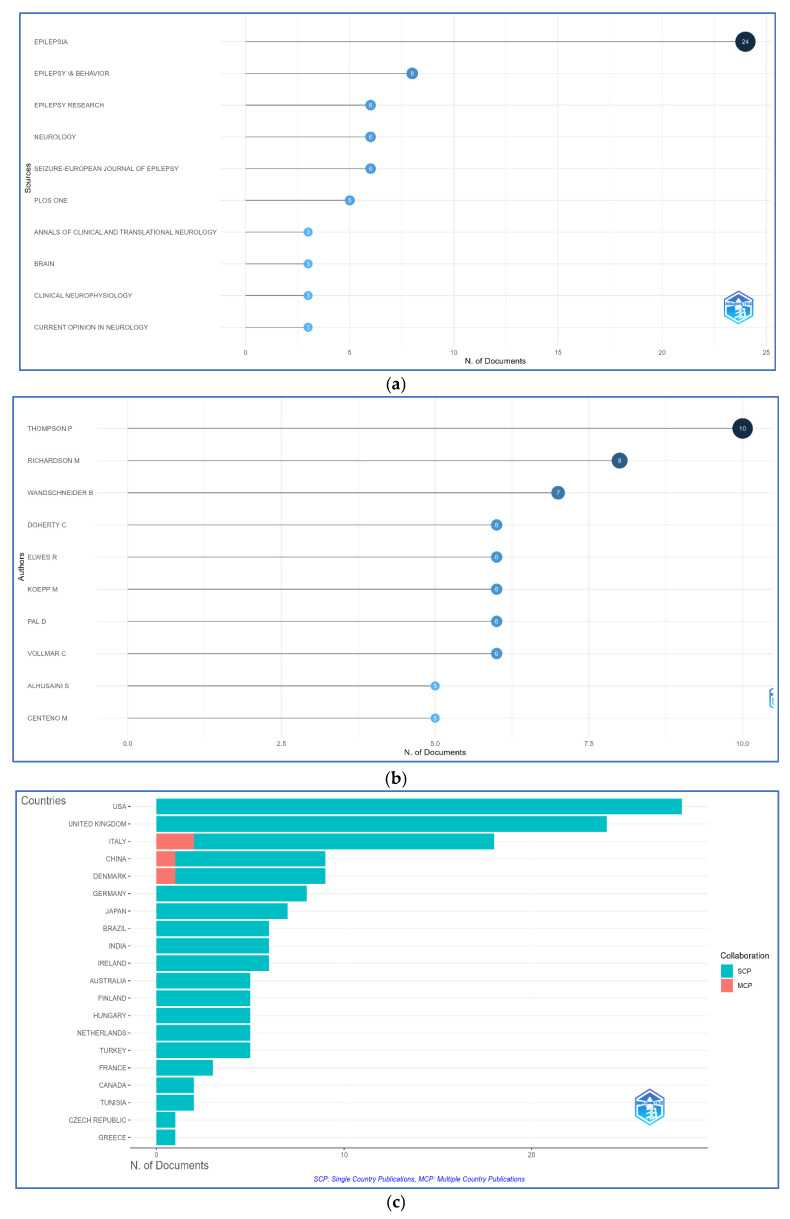
Research distributions and authorship showing (**a**) most producing sources, (**b**) most publishing authors, and (**c**) corresponding authors’ countries and collaboration.

The journal distribution reflected a clear publishing preference, with Epilepsia dominating with 24 papers, followed by other specialized journals (Epilepsy and Behavior, Epilepsy Research, and Seizure: European Journal of Epilepsy) and high-impact journals (Brain and Neurology), demonstrating the fundamental nature and clinical relevance of the research. PLOS ONE’s presence may reflect the growing trend toward open access (Figure 3a).

Author productivity highlighted Paul Thompson as the most prolific author (ten publications, 5.9%), followed by Mark Richardson (eight publications, 4.7%) and Britta Wandschneider (seven publications, 4.1%) (Figure 3b).

The United States, the United Kingdom, and Italy produced the most publications, with significant contributions also coming from China, Denmark, Germany, and several other countries. Most studies were single-country publications (SCPs), with limited multi-country publications (MCPs) primarily between Italy and China, indicating concentrated research efforts and limited global cooperation (Figure 3c).

In the topic trend analysis, classification emerged as the most prominent recent theme (2020–2024). Genetic research has shown strong momentum, with mutations exhibiting a high frequency and recent emergence. The concept of endophenotypes gained prominence around 2017–2018, while syndrome-specific research exhibited temporal clustering: temporal-lobe epilepsy (TLE) dominated early research (2013–2016), followed by juvenile myoclonic epilepsy (JME) as the primary focus from 2014 to 2020. Structural markers, including hippocampus atrophy, gained prominence during 2015–2018, indicating growing interest in brain region-specific endophenotypes.

**Figure 4:**
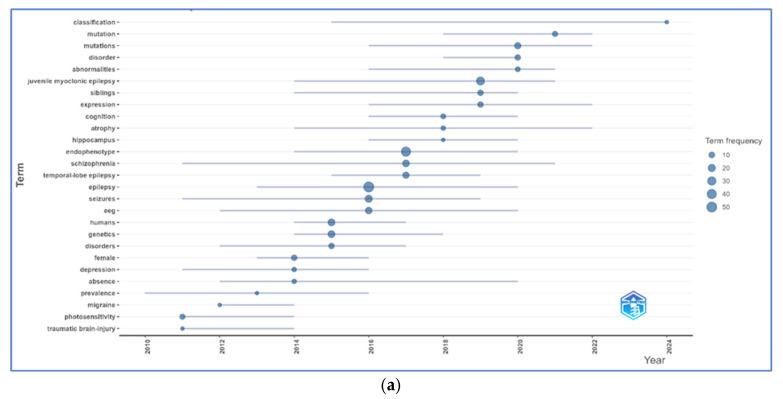

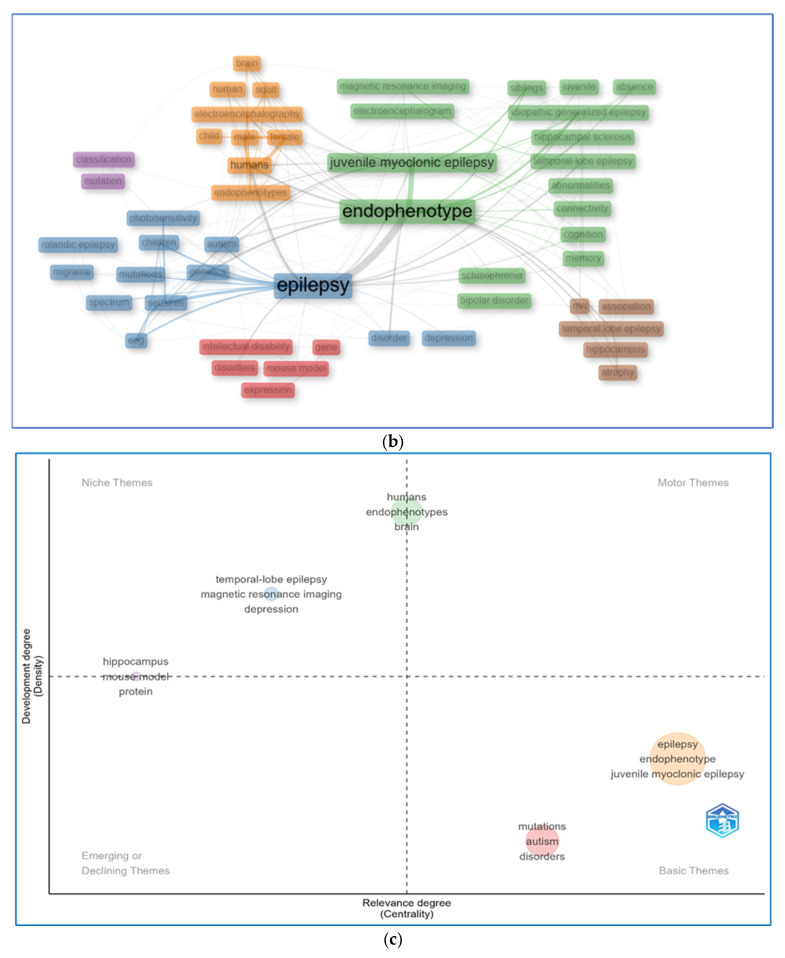
Network visualization of keyword co-occurrence and research focus evolution and theme (**a**) topic trend, (**b**) co-occurrence network cluster, and (**c**) thematic map.

Electroencephalography (EEG) maintained a consistent presence from 2011 to 2018, while genetics showed increasing prominence throughout the same period. Expression emerged as a molecular focus post-2016, complementing the recent genetic emphasis. The prominence of schizophrenia indicates successful expansion to other neuropsychiatric conditions. Core terms, including epilepsy and seizures, maintained steady baseline activity throughout the period. Early themes (2010–2015), including photosensitivity, traumatic brain injury, and migraine, showed lower intensity (Figure 4a).

Keyword co-occurrence analysis identified three primary thematic clusters: the first cluster centered on specific epilepsy syndromes (JME, TLE, idiopathic generalized epilepsy (IGE; also termed genetic generalized epilepsy or GGE)), the second cluster encompassed methodological approaches (EEG, magnetic resonance imaging (MRI), functional MRI (fMRI), genetic analysis), and the third cluster focused on neuropsychological and psychiatric dimensions, (cognitive dysfunction, psychiatric comorbidities, and behavioral phenotypes) (Figure 4b).

The thematic analysis plot illustrates the relationship between research topic centrality and development density across four distinct quadrants: motor themes (top-right) such as epilepsy and JME (mature and central status), basic themes (bottom-right) including mutations and autism (relevant), niche themes (top-left) covering TLE, MRI, and depression (specialized topics), and emerging/declining themes (bottom-left) representing nascent or waning interests (Figure 4c).

### 3.2. Methodological Quality and Evidence Certainty

All 53 included studies received complete NOS assessments. The methodological quality was consistently high, with 52 studies (98.1%) rated as high quality (7–9 stars) and only 1 study (1.9%) rated as medium quality (6.5/9 stars). No studies received low-quality ratings. Risk of bias assessments revealed 36 studies (67.9%) with a low risk of bias, 13 studies (24.5%) with a low-to-moderate risk, 3 studies (5.8%) with a minimal risk, and 1 study (1.9%) [37] with a moderate risk.

Inter-rater agreement for NOS quality assessment was excellent, with 94.3% of studies (50/53) receiving identical scores from both reviewers. The three studies with discrepant ratings [37,38,39] showed only one-point differences (seven vs. eight stars), and consensus was achieved through discussion.

GRADE-style assessments revealed variation across endophenotype classes, ranging from high to moderate certainty (Supplementary Table S5). Neuroimaging endophenotypes demonstrated HIGH certainty evidence (18 studies; 77.8% validation rate), supported by strong family study designs and consistent findings, though limited by predominantly European samples and equipment costs. Genetic/molecular markers demonstrated moderate to high certainty (six studies, 83.3% validation rate) with direct heritability evidence, but limited population diversity.

Cognitive, electrophysiological, psychiatric, and clinical phenotyping endophenotypes all demonstrated moderate certainty evidence. Cognitive markers (ten studies, 60% validation rate) benefited from validated, accessible tests; however, they showed variability in assessment batteries. Electrophysiological markers (eleven studies, 27.3% validation rate) were limited by poor standardization despite being non-invasive and cost-effective. Psychiatric (three studies) and clinical phenotyping (five studies) markers showed promise but required larger evidence bases.

Clinical readiness assessments indicated that neuroimaging, genetic/molecular, and cognitive endophenotypes showed moderate- high potential for near-term clinical implementation, while electrophysiological, psychiatric, and clinical phenotyping markers required additional validation and standardization (clinical readiness: low to low-moderate).

### 3.3. Systematic Review Results

Fifty-three studies met the inclusion criteria for detailed qualitative analysis, focusing on the validation of endophenotypes in epilepsy (Supplementary Table S1).

**Figure 5:**
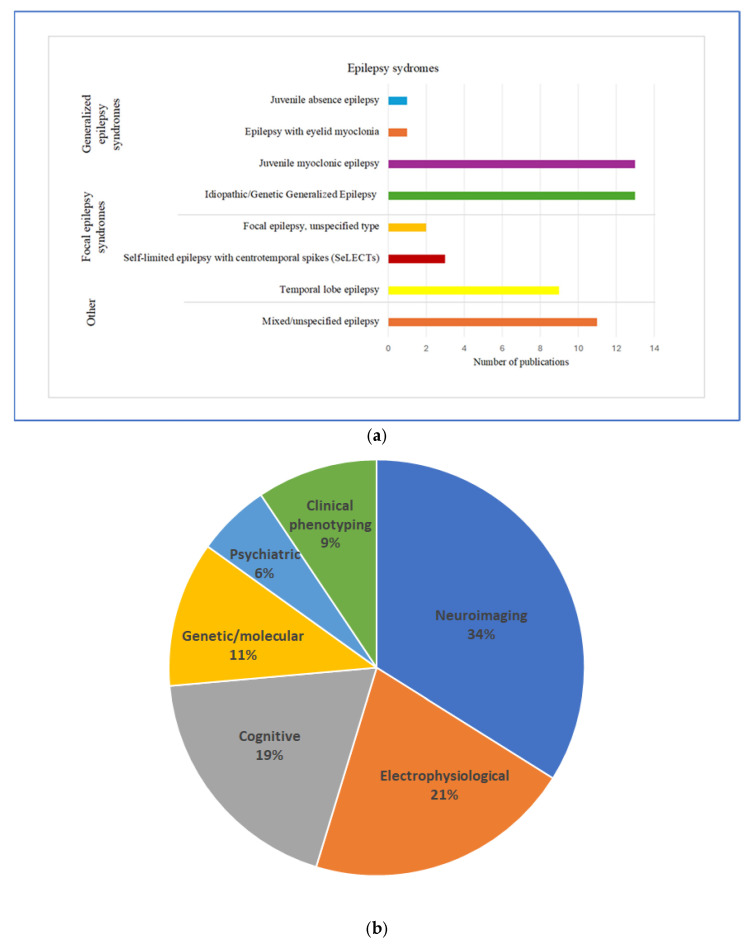
Endophenotypes in epilepsy research: (**a**) most studied epilepsy syndromes and (**b**) distribution of endophenotype categories.

Generalized epilepsy syndromes accounted for the majority (52.8%, n = 28), with idiopathic/genetic generalized epilepsy (IGE/GGE) and JME each represented in 13 studies (24.5% each), and single studies focusing on epilepsy with eyelid myoclonia (EEM) and juvenile absence epilepsy (JAE). Focal epilepsy (FE) constituted 26.4% (n = 14), primarily TLE (17%, n = 9), with three studies on self-limited epilepsy with centro-temporal spikes (SeLECTS) (formerly Rolandic epilepsy, 5.7%) and two that reported FE without further specification (3.8%). Mixed or unspecified epilepsy populations represented 20.8% (n = 11) (Figure 5a).

The systematic review identified six major categories of endophenotypes investigated in epilepsy research (Figure 5b).

- Neuroimaging (33.9%, n = 18) was the most frequent method, featuring fMRI (15.1%, n = 8) [40], volumetric and morphometric studies (9.4%, n = 5), magnetoencephalography (MEG) (3.8%, n = 2), diffusion tensor imaging (DTI) (1.9%, n = 1), quantitative MRI mapping (1.9%, n = 1) and conventional MRI with machine learning validation (1.9%, n = 1). JME and TLE were the primary syndromes studied with these methods, each represented in 6 studies (11.3%) [41,42,43,44].
  In JME, abnormalities included cortical thickness, subcortical volume loss [45], motor network hyperactivation [46] and co-activation [47], as well as abnormal hippocampal (fMRI) [48] and prefrontal/cingulate structures (morphometric studies) [49].
  TLE studies highlighted hippocampal functional disruptions [50,51], volumetric changes [52], morphological alterations [53,54], and white matter abnormalities [55].
- Electrophysiological endophenotypes (20.8%, n = 11) were the second most commonly used methodology, predominantly using EEG in seven studies (13.2%), and combined modalities like magnetic evoked potential (MEP) with EEG or transcranial magnetic stimulation (TMS), as well as single studies of auditory and visual evoked potentials (each 1.9%, n = 1) [56,57,58]. Primarily focused on generalized epilepsy syndromes (IGE/GGE n = 5, 9.4%; JME n = 2, 3.8%), key findings were MEP polyphasia [59], elevated resting-state EEG theta activity [60], and altered network connectivity [61,62,63,64,65].
- Cognitive endophenotypes (18.9%, n = 10) applied neurocognitive assessments, identifying general cognitive impairment (n = 4) [66,67,68,69], executive dysfunction (n = 3) [70,71,72], language, and attention deficits mostly in generalized epilepsy cohorts [69,73,74,75,76].
- Genetic/molecular endophenotypes (11.3%, n = 6) explored modifier genes (e.g., GABRG2, SCN1A), polygenic risk scores, and epigenetic markers relevant to epilepsy risk and treatment response. These typically involved mixed or unspecified epilepsy types [77,78,79,80,81].
- Psychiatric endophenotypes (5.7%, n = 3) investigated interictal dysphoric disorder [82], anxiety [39], and psychiatric comorbidities [38].
- Clinical phenotyping (9.4%, n = 5) included long-term studies on syndrome characterization [83], comorbidities [84], familial/sporadic distinctions [85], and prodromal trait identification [86].

### 3.4. Validation Performance

Detailed validation scores for each study, as evaluated against both frameworks, are presented in Supplementary Table S1. The complete scoring codebook and operational definitions are provided in Supplementary Table S2.

Under Endophenotype 2.0 criteria, 58.5% (n = 31/53) of studies achieved perfect validation scores (maximum 3 points out of 3), whereas with traditional criteria, only 43.4% of studies (maximum of 5 points out of 5) did (Supplementary Table S1). Neuroimaging endophenotypes demonstrated the highest success rate, with 77.8% achieving perfect scores (n = 4/5). fMRI led to an overall success rate of 87.5% (n = 7/8) and 100% success in family-based studies. Multimodal EEG–fMRI studies uniformly scored perfectly.

Cognitive endophenotypes had moderate validation success (60%), while electrophysiological endophenotypes showed lower success (27.3%). Genetic/molecular endophenotypes performed well under the updated criteria (83.3%), whereas none reached perfect scores under traditional measures.

The presence of a family-based study design was the strongest predictor of validation success: 96% (24/25) of studies incorporating family data achieved perfect scores, compared to 25% (7/28) without such data.

Genetic mediation was confirmed in 52.8% of studies, with direct genetic analyses and family co-segregation substantially outperforming population-based inference. High-performing studies often employed prospective designs, large sample sizes, and multi-site validations [77,87].

### 3.5. Inter-Rater Reliability Assessment Results

For the Gottesman and Gould criteria, rater one scores ranged from zero to five, while rater two scores ranged from one to five. The unweighted Cohen’s kappa indicated a moderate level of agreement (k = 0.543, Z = 6.91, *p* = 4.68 × 10^−12^). The linear weighted kappa showed substantial agreement (k = 0.728, Z = 6.77, *p* = 1.27 × 10^−11^). Quadratic weight produced an almost perfect agreement estimate (k = 0.817, Z = 5.96, *p* = 2.50 × 10^−9^). Raters provided identical scores in 36 out of 53 cases, corresponding to a simple agreement rate of 67.9%. For the Endophenotype 2.0 criteria, rater one’s scores ranged from one to three, and rater two’s scores ranged from two to three. The unweighted Cohen’s kappa indicated a substantial level of agreement between raters (k = 0.696, Z = 5.54, *p* = 2.97 × 10^−8^). The linear weighted kappa demonstrated substantial agreement (k = 0.716, Z = 5.84, *p* = 5.30 × 10^−9^). With quadratic weights, the reliability remained within the substantial agreement range (k = 0.749, Z = 5.64, *p* = 1.77 × 10^−8^). The raters assigned identical scores to 45 out of 53 cases, corresponding to a simple agreement rate of 84.91%.

### 3.6. Meta-Analysis of Validation Proportions and Meta-Regression Results

The pooled odds ratio was 2.23 (95% CI: 0.52–9.52; *p* = 0.278), indicating that the overall association was not statistically significant, and the odds of achieving a perfect score did not differ reliably between groups. There was considerable heterogeneity (I^2^ = 93.1%; τ^2^ = 27.00; Q (52) = 750.32, *p* < 0.0001). The overall null effect is driven by substantial heterogeneity and a design-type interaction; therefore, the subgroup/meta-regression findings should be treated as the primary inferential results.

Meta-regression analysis of study year shows that it did not significantly predict odds of perfect scoring (β = −0.044, *p* = 0.781). The OR per one-year increase was 0.96 (95% CI: 0.70–1.30), indicating no temporal trend. The sample size was not a significant moderator (β = −0.800, *p* = 0.272), and modality showed a borderline overall effect (QM (5) = 9.91, *p* = 0.078); however, no single modality differed significantly from the clinical reference category. Genetic (*p* = 0.058) and neuroimaging studies (*p* = 0.083) showed trends toward higher odds of perfect scoring, consistent with the descriptive results.

**Figure 6:**
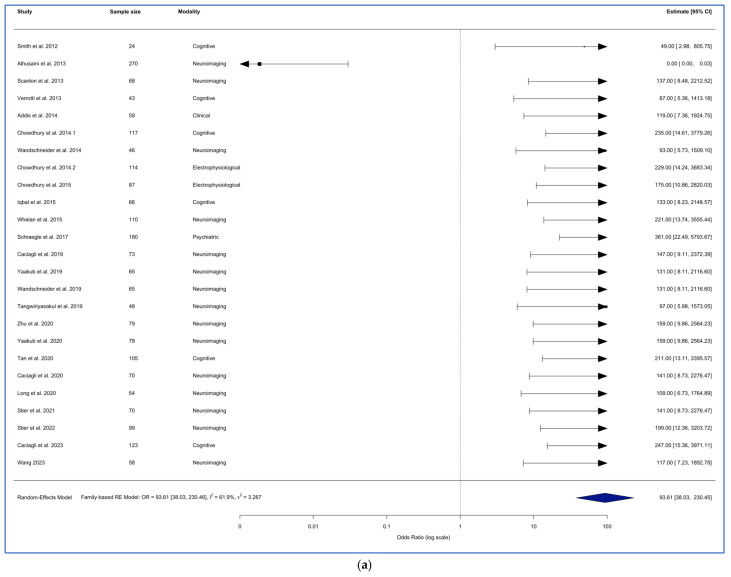

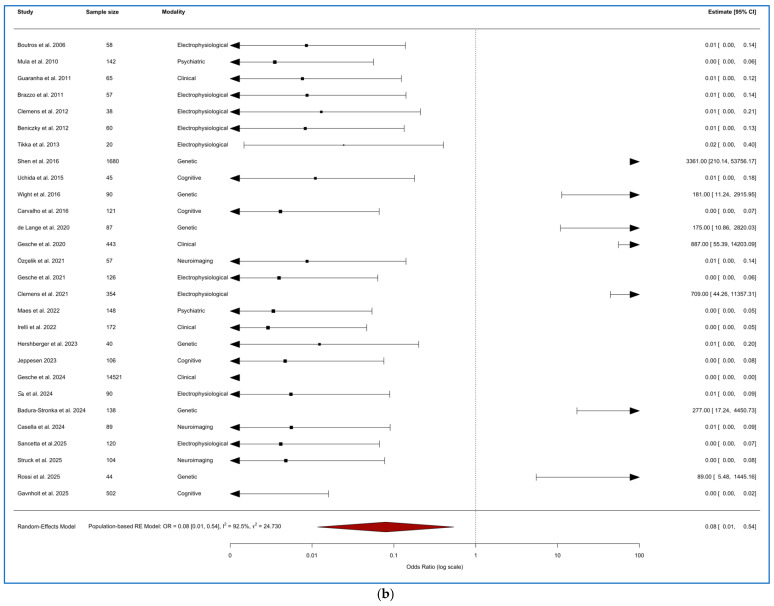
Forest plot of the random effects meta-analysis for (**a**) family-based study and (**b**) population-based study [37,38,39,40,41,42,43,44,45,46,47,48,49,50,51,52,53,54,55,56,57,58,59,60,61,62,63,64,65,66,67,68,69,70,71,72,73,74,75,76,77,78,79,80,81,82,83,84,85,86,87,88,89].

Study design was the strongest and most significant predictor. A subgroup analysis by study design showed that the random-effects model for family-based designs (k = 25) yielded a significant pooled effect (log-odds = 4.54, SE = 0.46, *p* < 0.0001). This corresponds to a pooled odds ratio (OR) of 93.62 (95% CI: 38.03–230.46). Although the direction of effect was strongly positive, the analysis showed moderately high heterogeneity (τ^2^ = 3.27; I^2^ = 61.86%; Q = 63.00, *p* < 0.0001) (Figure 6a).

In contrast, population-based studies (k = 28) produced a significant negative pooled effect (log-odds = –2.53, SE = 0.98, *p* = 0.0096), corresponding to a pooled OR of 0.079 (95% CI: 0.012–0.54). This suggests an opposite direction of effect relative to family-based designs. However, this subgroup showed extreme heterogeneity (τ^2^ = 24.73; I^2^ = 92.47%; Q = 359.65, *p* < 0.0001) (Figure 6b), indicating substantial inconsistency in effect sizes, likely reflecting methodological diversity among population-based studies.

A mixed-effects meta-regression confirmed a statistically significant difference between family-based and population-based designs (QM = 39.69, *p* < 0.0001). Population-based studies showed a lower effect than family-based studies, with a between-group log-odds difference of –7.07, corresponding to an OR of 0.001 (95% CI: 0–0.008). Study design accounted for 45.8% of the total heterogeneity, indicating that design type is a major determinant of effect variation across the literature.

### 3.7. Sensitivity Analysis Results

The leave-one-out analysis demonstrated that excluding any single study did not alter the pooled effect size, which remained within a restricted interval (β ≈ 0.66–1.02), and confidence intervals consistently crossed the null value. Across all iterations, heterogeneity statistics showed minimal fluctuation (I^2^ ≈ 92.6–93.2%), and τ^2^ remained high, indicating that the considerable between-study variability was not driven by an isolated study. Q-statistics remained significant in all cases (Q ≈ 687–746, *p* < 0.001), confirming persistent and substantial heterogeneity.

**Figure 7:**
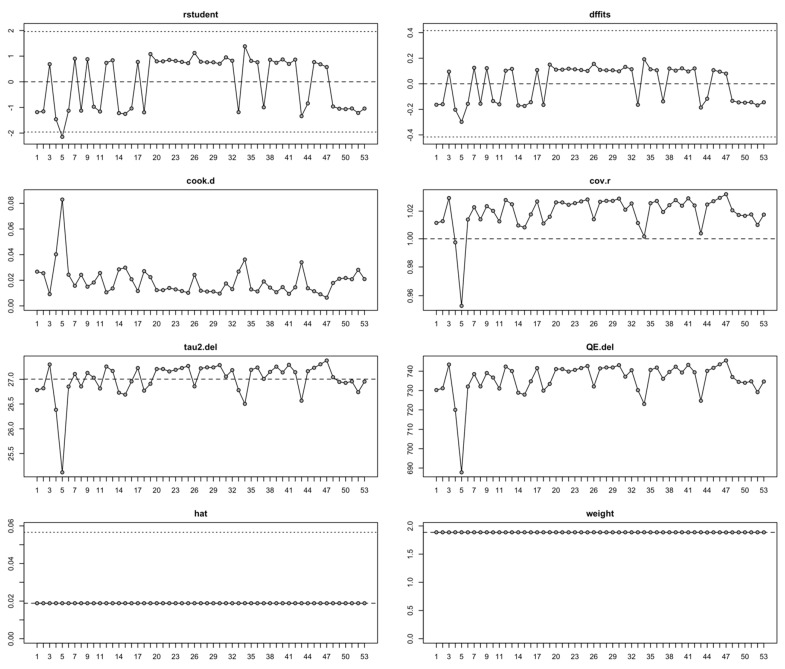
Influence diagnostics plot of the meta-analysis, showing how each of the 53 studies affects various aspects of the overall results.

Influence diagnostics, evaluated across eight components (Figure 7), demonstrated that the meta-analytic results were generally stable. Most studies showed standardized residuals within the ±2 boundary, indicating good conformity with model expectations. Study 5 (Gesche et al., 2020 [85]) displayed a large negative residual of approximately –2, indicating that its effect size was significantly lower than predicted by the model. Leverage (hat values) remained flat at ~0.02 for all studies, consistent with the expected equal leverage in a dataset of 53 studies (1/n ≈ 0.019). Study weights were likewise uniform (~1.9% per study), indicating an equal-weight or nearly equal-variance contribution to the pooled model; importantly, no single study was given disproportionate influence due to extreme precision.

### 3.8. Machine Learning Prediction of Validation Score Using a Random Forest Model Results

Across the 53 included studies, sample sizes ranged from 20 to 14,521 (median = 87), with balanced representation of family-based (47%) and population-based (53%) designs. Validation scores were distributed as three (58.5%), two (35.8%), and one (5.7%), with 31 studies (58.5%) achieving a perfect score.

**Figure 8:**
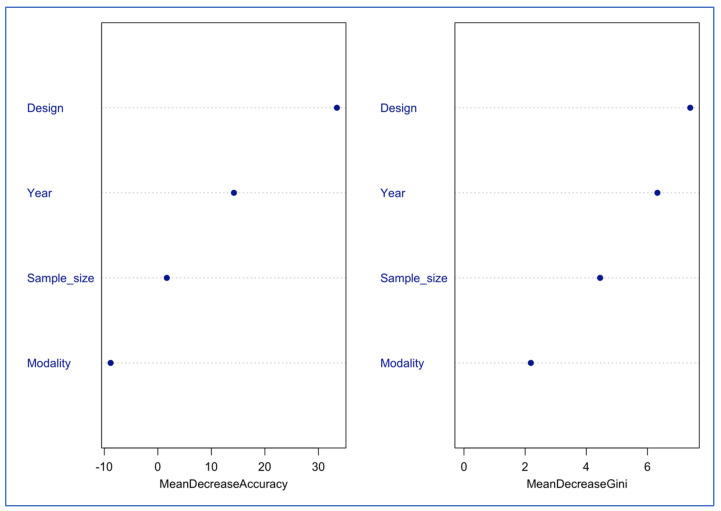
Variable importance for predicting validation scores 1 to 3.

For the multiclass prediction of validation scores one (n = 3), two (n = 19), and three (n = 31), the random forest classifier trained on 70% (n = 39) of the dataset achieved an out-of-bag error of 20.5%, indicating moderate discriminative performance. Misclassification was driven primarily by score one, which had no correctly predicted instances in the training OOB confusion matrix, reflecting the rarity of this class (only three studies). On the test set (n = 14), the model achieved an accuracy of 0.86 (95% CI: 0.57–0.98) and a Kappa of 0.71, indicating substantial agreement beyond chance. The classifier performed best for score three (sensitivity = 0.78; specificity = 1.00), with score one being perfectly detected (sensitivity = 1.00) and exhibiting a high ability to detect false positives (specificity = 0.78). Score one was not detected in the test set due to its very low prevalence. Study design was the strongest predictor of the assigned validation score, followed by year of publication and sample size. Modality showed minimal predictive contribution (Figure 8).

**Figure 9:**
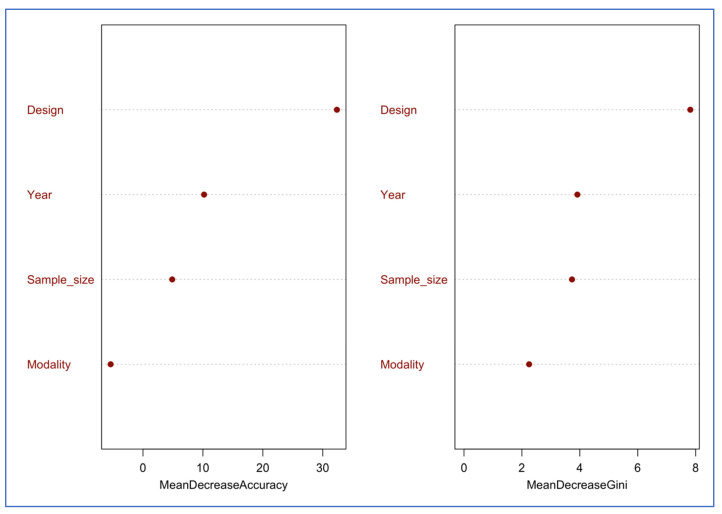
Variable importance for predicting a perfect score.

The binary random forest classifier demonstrated stronger predictive performance than the multiclass model. The out-of-bag error was 10.5%, and the training set showed high sensitivity for predicting perfect-scoring studies (0.86) and excellent specificity for non-perfect-scoring studies (0.94). On the test set, the classifier achieved an accuracy of 0.87, a sensitivity of 1.00 (all non-perfect studies were correctly identified), a specificity of 0.78 (the majority of perfect-score studies were correctly detected), a precision of 0.75, an F1-score of 0.86, and a balanced accuracy of 0.89. The ROC analysis demonstrated excellent discrimination, with an AUC of 0.94 (95% CI: 0.84–1.00). Study design again emerged as the dominant predictor, followed by year and sample size; modality contributed very little (Figure 9).

**Figure 10:**
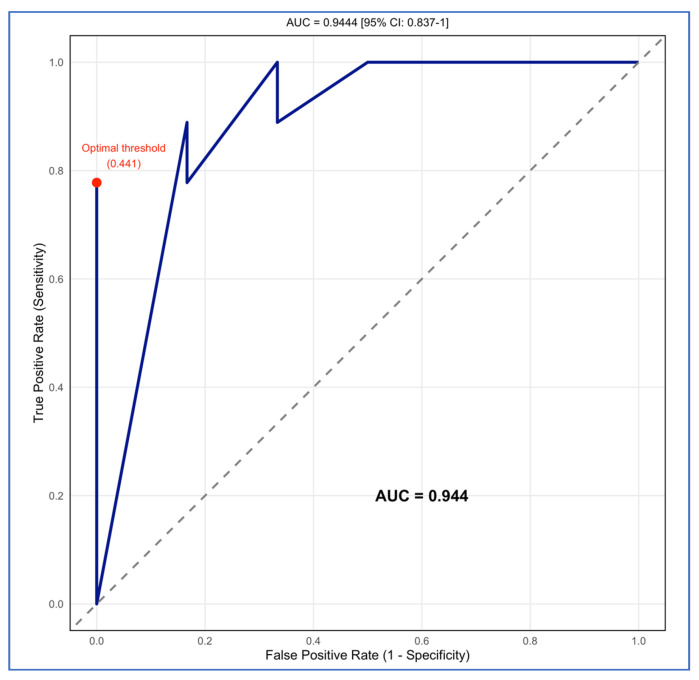
The ROC curve of the random forest model for a perfect validation score.

The model exhibited excellent discriminative ability, with a test-set AUC of 0.9444 (Figure 10). Models with a value greater than 0.9 are considered excellent [90,91]. The optimal threshold (Youden’s Index = 0.441) produced a sensitivity of 0.7778 and a specificity of 1.0, demonstrating high balanced performance at the optimal cut-point.

**Figure 11:**
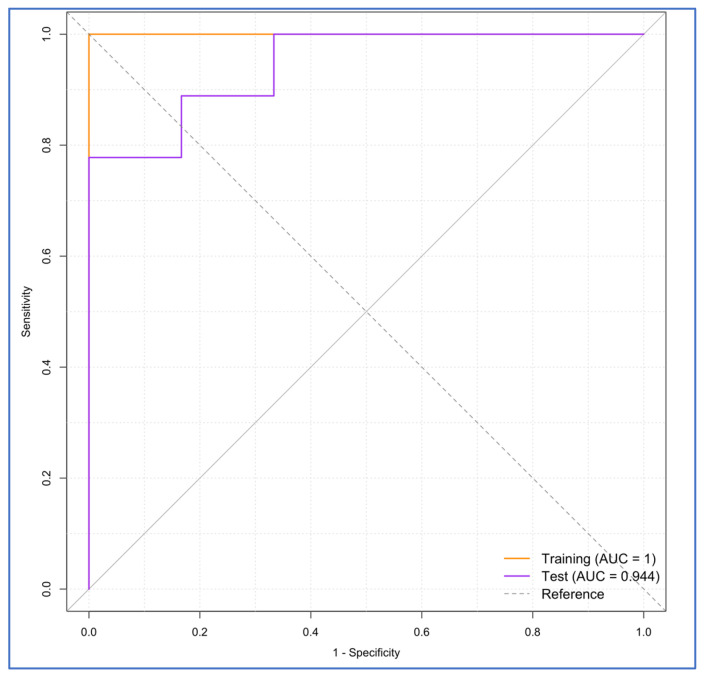
The ROC curve of the overfitting assessment using the training vs. the test set.

Overfitting assessment indicated good generalization. The training AUC was 1.0, and the test AUC was 0.9444 (Figure 11), resulting in a slight and acceptable difference (0.0556).

## 4. Discussion

This study represents the first comprehensive evaluation of epilepsy endophenotype research, analyzing 169 publications spanning a two-decade period. It reveals a transformation from basic EEG markers to sophisticated precision medicine tools, positioning epilepsy endophenotype research as a paradigm shift toward personalized healthcare approaches.

### 4.1. Field Evolution and Research Landscape

Epilepsy endophenotype research has evolved through three phases: emerging (2001–2005), establishing foundational concepts; growth (2006–2010), developing methodological frameworks; and expansion (2015–2025), which has dramatically increased the number of validated biomarkers and clinical applications. This acceleration parallels advances in high-resolution neuroimaging, next-generation sequencing capabilities, and computational approaches (Figure 2b,c) [92,93].

The discipline has matured from academic validation studies to clinically relevant biomarkers. Neuroimaging endophenotypes, particularly fMRI studies, demonstrate reproducible and clinically implementable tools with the possibility of integration into healthcare. The reliable replication of anomalies in motor and cognitive networks across different research groups provides a strong evidence base for clinical protocols [46,47,48,49,70].

However, the concerning finding that only 4.1% of studies involved international collaboration highlights a critical limitation affecting global applicability. The dominance of European and North American research sites limits the precision of medicine applications across diverse populations, where genetic architecture variations could affect biomarker performance, despite epilepsy’s global burden. Establishing worldwide collaboration networks ensures confirmed endophenotypes function consistently across diverse global populations [94,95].

The clinical implementation of biomarkers for epilepsy endophenotypes requires regulatory approval, integration into the healthcare system, and clinician training protocols, leveraging non-invasive and accessible assessment methods from successful biomarker implementations in other medical fields [96,97].

A notable limitation is the marked geographic concentration of endophenotype research. Only 4.1% of studies involved international collaboration between high-income and middle-income countries, with studies concentrated in Europe (45.3%), North America (26.4%), and East Asia (15.1%). This Euro-North American bias raises concerns about generalizability, particularly for genetic endophenotypes that may not translate across ancestry groups due to differences in allele frequencies and population-specific variants [98,99]. Similarly, neuroimaging and cognitive endophenotypes derived from Western populations may not account for neuroanatomical variation related to genetic ancestry, environmental exposures, or cultural differences in neurodevelopment [100]. Neuroimaging research broadly suffers from limited diversity, with only 10% of studies reporting race and 4% reporting ethnicity, and racial/ethnic minorities remain substantially underrepresented despite higher disease burden in these populations [100,101]. Future research should prioritize international consortia and the recruitment of diverse populations to ensure the equitable translation of endophenotype findings globally.

### 4.2. Validation Framework Revolution

Our comparative analysis revealed a critical paradigm shift where the Endophenotype 2.0 framework’s superior performance (58.5% perfect validation vs. 43.4% traditional) reflects the field’s evolution toward practical clinical utility over theoretical accuracy. The transformation addresses the limitations of conventional validation criteria, which often exclude clinically valuable biomarkers due to overly restrictive requirements [4,6]. The development of an explicit scoring codebook with concrete examples from included studies (Supplementary Table S2) represents a methodological contribution that enhances reproducibility and enables other research teams to apply consistent validation standards across different endophenotype domains and neurological conditions.

Random-effects meta-analysis revealed that Endophenotype 2.0 criteria yielded a pooled perfect validation rate of 54.7% compared to 15.1% under classical criteria, demonstrating a nearly fourfold increase in validation success through the accommodation of state-dependent markers and modernized heritability evidence.

Meta-regression identified family-based study design as the strongest predictor of validation success, underscoring the foundation of the endophenotype concept in familial aggregation. Neuroimaging markers showed the most significant improvement under Endophenotype 2.0, followed by genetic/molecular markers (83.3% vs. 33.3%; OR = 10.0). In contrast, electrophysiological and cognitive endophenotypes showed more modest gains, suggesting that measurement reliability remains a limiting factor.

Substantial heterogeneity (I^2^ = 68%) was partially explained by sample size and follow-up duration, with studies involving more than 100 participants demonstrating more consistent validation. These findings support the adoption of Endophenotype 2.0 as the preferred framework and the prioritization of family-based designs and large-scale cohorts for future endophenotype research.

Family-based designs demonstrated a strong and consistent association with the examined endophenotype, which was primarily composed of neuroimaging. In contrast, population-based studies showed an inverse effect with substantial heterogeneity, underscoring the study design as a critical source of variability in epilepsy endophenotype research. Family-based studies are crucial in identifying endophenotypes in neurological disorders, as they encompass the heritability and cosegregation criteria that are key differentiators of endophenotypes and biomarkers [102].

All endophenotypes are biomarkers, but not all biomarkers are endophenotypes [4,103]. While studies in population-based, unrelated individuals remain valuable, Glahn and Blangero (2011) highlight that biomarkers from such studies may need to be validated using family data to be definitively confirmed as true endophenotypes [102]. Family-based studies also provide a single representation for capturing and assessing all the endophenotype criteria concurrently [104].

The key difference between validation frameworks is their approach to genetic evidence. Traditional criteria require formal heritability estimates, and extensive family co-segregation data have proven incompatible with population-based genomics, polygenic risk scores, and genome-wide association studies, which represent the current standard for biomarker development [4,105]. The Endophenotype 2.0 framework’s pragmatic approach—requiring reliable measurement, disease association, and genetic mediation—captures clinically meaningful biomarkers while maintaining scientific rigor [6].

The framework evolution also reflects shifting views on what makes an effective biomarker. Because the old criteria focused on state independence, they did not incorporate potentially instructive signs that vary with disease activity or treatment. Yet from a precision medicine perspective, markers predicting treatment response or disease progression might be more valuable. Endophenotype 2.0 creates new opportunities for developing biomarkers by allowing for state-dependent markers [96].

A significant development in the Endophenotype 2.0 framework is the inclusion of state-dependent markers alongside traditional state-independent traits, expanding the endophenotype concept beyond classical criteria [6]. Traditionally, endophenotypes were expected to remain constant regardless of symptomatic status, which limited their applicability in conditions like epilepsy, where factors such as seizure activity introduce state-dependent variability [4]. Endophenotype 2.0 enables the identification of state-dependent markers with genetic mediation, recognizing that traits can vary according to disease activity or treatment. The implications for translational research are substantial, as state-dependent endophenotypes can serve as valuable biomarkers for treatment response. For example, changes in resting-state network connectivity post-epilepsy surgery could indicate treatment efficacy, thereby linking mechanistic biomarkers to clinically relevant applications and enhancing the translational potential of epilepsy endophenotype research [9].

However, we should not interpret our findings as suggesting that traditional criteria lack value. The exceptional performance of family-based designs (with 96% perfect validation) demonstrates that genetic context remains crucial. This finding suggests the use of complementary rather than competing methodologies: family studies for initial discovery and validation, and population studies for replication and the possibility of clinical translation [106,107].

### 4.3. Promising Endophenotypes

The validation success rates across endophenotype categories demonstrate differential readiness for clinical implementation, with neuroimaging markers having the most significant potential for improvement. The fMRI studies achieving 100% validation success in family-based designs, particularly those examining motor and cognitive networks, provide objective and reproducible biomarkers for these areas. Detecting these abnormalities in unaffected relatives can help assess presymptomatic risk and implement preventive interventions [46,47,51,88].

Despite modest validation rates (60%), cognitive endophenotypes offer unique advantages due to their accessibility—non-invasive, inexpensive, and repeatable assessments suitable for widespread implementation. The constant observation that executive dysfunction is found in all epilepsy types points to common neural substrates that may be targeted for treatment. Additionally, cognitive signs frequently appear before the onset of seizures, offering opportunities for early intervention [66,67,69,70].

The remarkable improvement in genetic/molecular endophenotypes under Endophenotype 2.0 criteria (83.3% vs. 0% perfect validation) highlights the field’s readiness for precision medicine approaches. Comprehensive genetic panels incorporating validated endophenotypes could become routine diagnostic tools, enabling clinicians to select appropriate medications, determine treatment eligibility, and achieve faster, more accurate diagnoses [77,87,108,109,110,111,112].

### 4.4. Transdiagnostic Implications

Our network analysis reveals that epilepsy endophenotypes may function as transdiagnostic markers between neurodevelopmental disorders, psychiatric conditions, and epilepsy endophenotypes, suggesting shared biological pathways that could revolutionize risk assessment and intervention approaches [39,66,67,69,89,113].

Instead of waiting for specific diagnostic criteria to be met, this transdiagnostic perspective enables clinicians to conduct a neurodevelopmental risk screening using validated endophenotype batteries, even before symptoms appear. This preventive approach suggests novel therapeutic targets, with medications targeting endophenotypes rather than diagnoses. Drug development could shift from disorder-specific to mechanism-specific approaches [96,109].

Our findings position epilepsy endophenotypes within the broader landscape of transdiagnostic neurodevelopmental markers. High-performing endophenotypes identified in this review—particularly neuroimaging (cortical thickness, surface area, and functional connectivity), cognitive (executive dysfunction and attention deficits), and genetic/molecular markers—show a remarkable overlap with biomarkers validated across other psychiatric and neurodevelopmental conditions [10,11,114].

Cortical structural alterations, one of the strongest endophenotype classes in our analysis (with a 77.8% validation rate), parallel findings from large-scale developmental cohorts, which demonstrate that deviations from normative cortical development predict dimensional psychopathology across both internalizing and externalizing profiles [10,12].

Executive dysfunction and attention deficits—cognitive endophenotypes with 60% validation rates in our review—represent prototypical transdiagnostic markers observed across epilepsy, ADHD, autism spectrum disorder, and mood disorders [115,116].

Clinical implications of transdiagnostic endophenotypes. Recognition of shared endophenotypes across epilepsy and other neurodevelopmental conditions has several implications. First, screening batteries assessing cortical structure, executive function, and genetic risk could facilitate early identification of children at risk for multiple neurodevelopmental outcomes, not epilepsy alone [10]. Second, interventions targeting shared mechanisms (e.g., E/I balance modulation and cognitive remediation) may benefit comorbid presentations. Third, clinical trials could leverage transdiagnostic endophenotypes as intermediate outcomes, potentially increasing statistical power and generalizability across disorders. Future validation studies should explicitly test whether epilepsy endophenotypes predict outcomes across diagnostic boundaries and whether they respond to interventions in transdiagnostic samples.

### 4.5. Limitations and Future Directions

Several methodological considerations limit the generalizability of our findings. The bibliometric analysis was constrained by potential selection bias, as it relied on database coverage that may have over-represented English-language and high-impact journals. Publication bias, which favors positive findings, may inflate validation success rates [117].

Temporal limitations in cross-sectional research are significant, as different markers are informative at various stages of life. Following endophenotypes from childhood to maturity, longitudinal studies can provide crucial information about marker changes and disease course [6].

The systematic review faced challenges due to heterogeneity and methodological variation across studies, requiring interpretive judgment and potentially introducing bias. Additionally, the bibliometric emphasis on publication patterns may not align with the quality of systematic evidence.

An essential limitation of this review is that the majority of included studies were conducted in Europe and North America. This regional concentration limits the generalizability of our findings to global populations, given the potential variability in genetic backgrounds, healthcare systems, and environmental factors across different regions.

Recent advances in artificial intelligence (AI) have begun to significantly impact epilepsy research, particularly in the areas of imaging and biomarker identification. AI-driven approaches facilitate the analysis of complex neuroimaging data, enabling the more precise identification of epilepsy endophenotypes and aiding in the uncovering of underlying mechanisms. For example, Berger et al. (2025) provide a comprehensive overview of AI applications in epilepsy imaging, highlighting its potential to transform both clinical and research paradigms [118,119].

Future research priorities emerge clearly from our analysis. First, international collaborative networks should be established to ensure that findings generalize across populations. Second, integrated multimodal assessment batteries should be developed. Third, longitudinal studies are needed. Fourth, clinical translation studies are also necessary. Fifth, researchers need to include more diverse populations to understand epilepsy endophenotypes better worldwide. Finally, machine learning methods can be used to identify complex endophenotype patterns invisible to traditional analyses.

## 5. Conclusions

Our findings demonstrate that epilepsy endophenotype research has transitioned from experimental tools to clinically actionable biomarkers. The validation of biomarkers spanning neuroimaging, cognitive, and genetic domains provides multiple pathways for clinical implementation, from routine screening protocols to precision medicine applications.

Adopting more pragmatic validation frameworks, like Endophenotype 2.0, reflects the field’s emphasis on clinically relevant discoveries over purely academic validation. The superior performance of family-based designs underscores the continued importance of genetic context, even as population-based approaches become increasingly feasible.

Validated endophenotypes can provide measurable bridges between genes and disease, offering clinicians evidence-based tools for genetic counseling, early intervention, and personalized treatment strategies. The journey from genes to seizures becomes clearer with each validated endophenotype, bringing us closer to the promise of precision medicine: providing the proper treatment for the right patient at the right time.

## Figures and Tables

**Table 1:** The search strategy.

Database	Strategy
Web of Science Core Collection	TS = ((“endophenotype *” OR “intermediate phenotype *” OR “endophenotyp *”) AND (“epileps *” OR “seizure *” OR “epileptic *” OR “temporal lobe epilepsy” OR “TLE” OR “juvenile myoclonic epilepsy” OR “JME” OR “focal epilepsy” OR “focal seizure *” OR “generalized epilepsy” OR “generalized seizure *” OR “idiopathic generalized epilepsy” OR “IGE” OR “genetic generalized epilepsy” OR “GGE”))
Scopus	TITLE-ABS-KEY((“endophenotype *” OR “intermediate phenotype *” OR “endophenotyp *”) AND (“epileps *” OR “seizure *” OR “epileptic *” OR “temporal lobe epilepsy” OR “TLE” OR “juvenile myoclonic epilepsy” OR “JME” OR “focal epilepsy” OR “focal seizure *” OR “generalized epilepsy” OR “generalized seizure *” OR “idiopathic generalized epilepsy” OR “IGE” OR “genetic generalized epilepsy” OR “GGE”))

* in the table as indicating truncation of the search terms by the wildcard operator.

## Data Availability

All data and materials supporting this study are openly available in the Open Science Framework (OSF) repository at https://doi.org/10.17605/OSF.IO/QBY4F. The repository includes extraction sheets, validation scoring rubrics, Newcastle-Ottawa Scale assessments, GRADE evidence evaluations, R code for meta-analysis, and machine-readable tables of all included studies, along with their corresponding PMIDs/DOIs. Supplementary Tables S2–S5 are available in the OSF repository and as supplementary files with this publication.

## Supplementary Materials

The following supporting information can be downloaded at: https://www.mdpi.com/article/10.3390/brainsci15121275/s1, Table S1: Validation Scores for All 53 Studies Against Traditional (Gottesman and Gould, 2003) [4] and Endophenotype 2.0 (Liu and Gershon, 2024) [6] Frameworks; Table S2: Operational scoring codebook for Endophenotype validation frameworks; Table S3: Codes; Table S4: NOS; Table S5: GRADE-Style Certainty of Evidence Assessment by Endophenotype Class.

## Abbreviations

The following abbreviations are used in this manuscript:

AUC	Area under the curve
DFFITS	Difference in Fits
DTI	Diffusion tensor imaging
EEG	Electroencephalography
FE	Focal epilepsy
GRADE	Grading of Recommendations Assessment, Development and Evaluation
fMRI	Functional MRI
IGE	Idiopathic generalized epilepsy
IGE/GGE	Idiopathic/genetic generalized epilepsy
JME	Juvenile myoclonic epilepsy
MCPs	Multi-country publications
MEG	Magnetoencephalography
MEP	Magnetic evoked potential
MeSH	Medical Subject Headings
MRI	Magnetic resonance imaging
NOS	Newcastle-Ottawa Scale
OOB	Out-of-bag
OR	Odds ratio
PRISMA	Preferred Reporting Items for Systematic Reviews and Meta-Analyses
REML	Restricted maximum likelihood
ROC	Receiver operating characteristic
SCPs	Single-country publications
SeLECTS	Self-limited epilepsy with centro-temporal spikes
TLA	Three-letter acronym
TLE	Temporal-lobe epilepsy
TMS	Transcranial magnetic stimulation
WoSCC	Web of Science Core Collection
