# Supplementary material for: Endophenotype Research in Epilepsy Across Time
Source: Brain Sci. 2025 Nov 27;15(12):1275. doi: 10.3390/brainsci15121275 (PMC12730710; doi:10.3390/brainsci15121275)
Supplement: Supplementary file 1 [file brainsci-15-01275-s001.zip › Supplementary Table S1-Validation of Studies.pdf]

	Author, Year, Theme, Reference Number	Validation Score according to Gottesman & Gould (2003) Criteria	Score	Analysis According to Endophenotype 2.0 (Liu & Gershon, 2024)	Score	Primary Strength
1	Sancetta et al. (2025)<br>Altered neural avalanche spreading in DRE<br>[56]	-Association with illness: Shows clear avalanche spreading differences between DRE vs. non-DRE vs. HC<br>-Heritability: Not assessed<br>-Family Cosegregation: Not assessed<br>-State-Independence: Not assessed<br>-Higher frequency in unaffected relatives: Not assessed	1/5	-Reliable Measurement: Sophisticated EEG avalanche analysis, rigorous graph theory metrics, and validated statistical approaches<br>-Association with disease or treatment: Clear relationship between altered avalanche spreading and DRE condition with high discriminative accuracy<br>-Genetic Mediation: No direct heritability, family data or genetic analysis	2/3	Sophisticated EEG avalanche analysis
2	Struck et al. (2025)<br>JME Imaging Endophenotypes<br>[45]	-Association with illness: Demonstrates apparent structural and functional differences between JME patients and HC, including cortical thickness abnormalities and subcortical volume reductions<br>-Heritability: Not assessed<br>-Family Cosegregation: Not assessed<br>-State-Independence: Structural imaging measures (cortical thickness, subcortical volumes) persist independent of seizure activity<br>-Higher frequency in unaffected relatives: Not assessed	2/5	-Accurate Measurement: Sophisticated 3Tesla MRI with standardized protocols and machine learning validation<br>-Association with disease or treatment: Clear relationships between imaging phenotypes and JME pathophysiology, cognition, and EEG patterns<br>-Genetic Mediation: No direct heritability, family data or genetic analysis	2/3	Neuroimaging with machine learning validation
3	Rossi et al. (2025)<br>GABRG2 Loss- and Gain-of-Function Variants<br>[78]	-Association with illness: GABRG2 variants are strongly linked to the spectrum of epilepsy phenotypes<br>-Heritability: Yes<br>-Family Cosegregation: Family inheritance and segregation studies included	4/5	-Reliable Measurement: Systematic phenotype-genotype, functional validation<br>-Association with disease or treatment: Mutations correspond with phenotype spectrum and severity	3/3	Phenotype-genotype, functional validation

		-State-Independence: Variants present regardless of seizure state<br>-Higher frequency in unaffected relatives: Not assessed		-Genetic Mediation: Direct functional analysis showing gain-of-function vs loss-of-function effects		
4	Gavnholt et al. (2025) Clustering IGE patients' phenotypes [72]	-Association with illness: Executive dysfunction is clearly associated with IGE syndromes<br>-Heritability: Partially met -IGE itself is genetic, though the specific heritability has not been studied<br>-Family Cosegregation: Not assessed<br>-State-Independence: Variants present regardless of epilepsy type (JME, JAE, EGTCS) and seizure state<br>-Higher frequency in unaffected relatives: Not assessed	2/5	-Reliable Measurement: Comprehensive analysis of patients with neuropsychological screening and EEG<br>-Association with disease or treatment: Yes<br>-Genetic Mediation: No direct heritability, family data or genetic analysis	2/3	Cognitive endophenotype validation
5	Gesche et al. (2024) Prodromal Phase of IGE [86]	-Association with illness: Clear association with IGE diagnosis, illness, and prodrome<br>-Heritability: Not assessed<br>-Family Cosegregation: Not assessed<br>-State-Independence: Present before seizure onset<br>-Higher frequency in unaffected relatives: Not assessed	2/5	-Reliable Measurement: Hospital cohorts, validated phenotypes<br>-Association with disease or treatment: Strong association with IGE<br>-Genetic Mediation: No direct heritability, family data or genetic analysis	2/3	Comprehensive analysis of patients, clinical phenotyping
6	Asha et al. (2024) EEG microstate parameters in different epilepsy types [44]	-Association with illness: EEG microstate parameters statistically distinct in TLE, IGE vs. HC<br>-Heritability: No direct heritability estimates provided<br>-Family Cosegregation: Not assessed<br>-State-Independence: Microstate parameters were measured during resting-	2/5	-Accurate Measurement: EEG analysis and machine-learning-based classifier<br>-Association with disease or treatment: Microstate patterns reliably distinguish groups, correlate with neurocognitive impairment<br>-Genetic Mediation: No direct heritability, family data or genetic analysis	2/3	EEG analysis with machine learning validation

		state conditions independent of seizure activity or clinical state<br>-Higher frequency in unaffected relatives: Not assessed				
7	Badura-Stronka et al. (2024)<br>Polish epilepsy genetic study<br>[87]	-Association with illness: Yes<br>-Heritability: Not assessed<br>-Family Cosegregation: Not assessed<br>-State-Independence: Genetic variants persist regardless of the epilepsy type and seizure state<br>-Higher frequency in unaffected relatives: Not assessed	2/5	-Reliable measurement: Rigorous genetic testing and phenotyping<br>-Association with disease or treatment: Diagnostic yield is highest in developmental/epileptic encephalopathy and early onset<br>-Genetic Mediation: Strong focus on genetic causation for specific clinical traits	3/3	Rigorous genetic testing and phenotyping
8	Casella et al. (2024)<br>Cortical microstructure alterations in pediatric focal epilepsy<br>[54]	-Association with illness: Quantitative MRI mapping of microstructure<br>-Heritability: Not assessed<br>-Family Cosegregation: Not assessed<br>-State-Independence: The marker (altered qT1/qT2) is present regardless of clinical severity, seizure frequency, duration, or lesion presence<br>-Higher frequency in unaffected relatives: Not assessed	2/5	-Reliable measurement: Validated for microstructural measurement<br>-Association with disease or treatment: Yes<br>-Genetic Mediation: No direct heritability, family data or genetic analysis	2/3	Novel, Quantitative Imaging Biomarker Across Diverse Pediatric Focal Epilepsy
9	Caciagli et al. (2023)<br>Cognitive phenotype in JAE and their siblings<br>[66]	-Association with illness: Clear cognitive impairments in JAE pts vs. HC across attention/psychomotor speed, language, and executive function<br>-Heritability: Strong inference from shared traits between affected/unaffected family members<br>-Family Cosegregation: Key strength: Unaffected siblings showed similar language impairment as patients, demonstrating familial cosegregation	5/5	-Reliable Measurement: Comprehensive neuropsychological battery with standardized tests and proper statistical controls<br>-Association with disease or treatment: Cognitive deficits linked to JAE pathophysiology and functional outcomes<br>-Genetic Mediation: Shared patterns in siblings indicate genetic influence	3/3	Cognitive endophenotype validation, sibling study

		-State-Independence: Cognitive traits present in seizure-free patients<br>- Higher frequency in unaffected relatives: Siblings > HC				
10	Hershberger et al. (2023)<br>Molecular subtypes and<br>post-surgical seizure recurrence<br>[81]	-Association with illness: Molecular subtypes associated with seizure recurrence<br>-Heritability: Not assessed<br>-State-Independence: Partially met - Epilepsy surgery pts<br>-Family Cosegregation: No family studies<br>-Higher frequency in unaffected relatives: Not assessed	1/5	-Reliable Measurement: RNA sequencing with rigorous statistical methods<br>-Association with disease or treatment: Predicts surgical outcomes<br>-Genetic Mediation: No direct heritability, family data or genetic analysis	2/3	Molecular analysis
11	Jeppesen et al. (2023)<br>Neuropsychological phenotype in IGE<br>[73]	-Association with illness: IGE patients showed significant impairments in semantic fluency, the Purdue Pegboard test, and vocabulary compared to HC<br>-Heritability: No direct heritability estimates provided (references prior sibling studies)<br>-Family Cosegregation: Not assessed<br>-State-Independence: Profile independent of seizure control status and medication<br>-Higher frequency in unaffected relatives: Not assessed	2/5	-Reliable Measurement: Standardized battery, compared to Danish norms<br>-Association with disease or treatment: Clear associations with IGE across all subsyndromes (JME, JAE, GTCA), independent of treatment effects<br>-Genetic Mediation: No direct heritability, family data or genetic analysis	2/3	Validated neuropsychological assessment
12	Wang et al. (2023)<br>Functional network abnormalities in TLE and their siblings<br>[51]	-Association with illness: Network inefficiencies and reduced connectivity in pts<br>-Heritability: Same abnormalities in unaffected siblings<br>-Family Cosegregation: Network abnormalities co-segregate in patient–sibling pairs	5/5	-Reliable Measurement: Advanced task-based fMRI, graph theory, robust stats, reproducible effects<br>-Association with disease or treatment: Network topology disruption relevant for seizure risk	3/3	Sophisticated task-based fMRI study with family validation

		-State-Independence: Yes<br>-Higher frequency in unaffected relatives: Siblings > HC		-Genetic Mediation: Statistical analysis between patient-sibling pairs demonstrates genetic influence		
13	Stier et al. (2022) EEG/MEG synchrony, cortical thinning in GGE and their siblings<br>[62]	-Association with illness: GGE patients showed increased functional connectivity<br>-Heritability: Yes<br>-Family Cosegregation: Healthy siblings, free of epilepsy, showed intermediate abnormalities<br>-State-Independence: Siblings without seizure history display some of the imaging phenotypes<br>-Higher frequency in unaffected relatives: Siblings > HC	5/5	-Reliable Measurement: Sophisticated EEG/MEG synchrony, cortical thinning measurement<br>-Association with disease or treatment: Network hyperexcitability, altered development known to relate to GGE pathology<br>-Genetic Mediation: Shared patterns in siblings indicate genetic influence	3/3	Sophisticated EEG/MEG synchrony, cortical thinning measurement
14	Maes et al. (2022) Comorbid psychiatric disorders in TLE<br>[38]	-Association with illness: Demonstrates oxidative stress markers differences between TLE patients vs. HC<br>-Heritability: Not assessed<br>-Family Cosegregation: Not assessed<br>-State-Independence: Abnormalities exist independent of seizure control status and medication<br>-Higher frequency in unaffected relatives: Not assessed	2/5	-Reliable Measurement: Oxidative stress biomarker assessment<br>-Association with disease or treatment: Shows strong correlations between biomarker patterns and clinical severity<br>-Genetic Mediation: No direct heritability, family data or genetic analysis	2/3	Biomarker assessment, psychiatric evaluation
15	Irelli et al. (2022) Long-term seizure outcomes in EEM<br>[83]	-Association with illness: Demonstrates distinct clinical patterns and seizure outcomes in pts vs. HC<br>-Heritability: Yes<br>-Family Cosegregation: Family history data collected, showing 20.3% had first-degree relatives with epilepsy<br>-State-Independence: Not assessed	3/5	-Reliable Measurement: Comprehensive clinical assessment with rigorous statistical methods, including 2-step cluster analysis<br>-Association with disease or treatment: Clinical subtypes clearly associated with seizure outcomes and neuropsychiatric comorbidities<br>-Genetic Mediation: Partially met	2/3	Large multicenter cohort with extended follow-up

		-Higher frequency in unaffected relatives: Not assessed				
16	Ur-Özçelik et al. (2021)<br>JME Photosensitivity<br>[40]	-Association with illness: Demonstrates apparent functional connectivity differences between photosensitive JME pts vs. non-photosensitive pts and HC<br>-Heritability: Not assessed<br>-Family Cosegregation: Not met<br>-State-Independence: Not assessed<br>-Higher frequency in unaffected relatives: Not assessed	1/5	-Reliable Measurement: Rigorous resting-state fMRI methodology with proper preprocessing and statistical controls<br>-Association with disease or treatment: Functional connectivity alterations clearly linked to photosensitive seizure mechanisms in JME<br>-Genetic Mediation: No direct heritability, family data or genetic analysis	2/3	Sophisticated fMRI methodology
17	Stier et al. (2021)<br>MEG Network Heritability in GGE and their siblings<br>[41]	-Association with illness: GGE patients showed increased power and connectivity<br>-Heritability: High intraclass correlations indicate a strong genetic influence<br>-Family Cosegregation: Siblings showed intermediate levels between pts and HC<br>-State-Independence: Network alterations present during rest are independent of seizure activity; trials with generalized spike-wave discharges were systematically excluded<br>-Higher frequency in unaffected relatives: Siblings > HC	5/5	-Reliable Measurement: Sophisticated processing with rigorous artifact rejection<br>-Association with disease or treatment: Network alterations clearly linked to GGE pathophysiology, thalamocortical dysfunction, and GABA receptor function<br>-Genetic Mediation: Shared patterns in siblings indicate genetic influence	3/3	Multimodal integration (EEG+MEG+MRI)
18	Gesche et al. (2021)<br>MEP Polyphasia Study in IGE<br>[59]	-Association with illness: Higher MEP polyphasia in IGE patients<br>-Heritability: Not assessed<br>-Family Cosegregation: Not assessed<br>-State-Independence: MEP polyphasia present regardless of current seizure activity<br>-Higher frequency in unaffected relatives: Not assessed	2/5	-Reliable measurement: Standardized MEP recording protocols<br>-Association with disease or treatment: Partially met<br>-Genetic mediation: No direct heritability, family data or genetic analysis	1/3	Sophisticated MEP methodology

19	Clemens et al. (2021) Resting-state EEG theta activity study [60]	-Association with illness: Elevated resting-state EEG theta in both major epilepsy syndromes (IGE and FE), showing disease association<br>-Heritability: Stronger theta increase in patients with positive (especially 1st-degree) family history of epilepsy<br>-Family Cosegregation: Theta activity is higher in patients with positive family history (especially 1st-degree), but no sibling or unaffected relative data are available<br>-State-Independence: Resting state EEG<br>-Higher frequency in unaffected relatives: Not assessed	4/5	-Reliable Measurement: Standardized EEG<br>-Association with disease or treatment: Strong association with the genetic determination of epilepsy<br>-Genetic Mediation: Strong association with genetic loading and positive family history	3/3	Study with drug-naive, newly diagnosed IGE and FE patients
20	Zhu et al. (2020) SCN8A Variant Family Neuroimaging Study [88]	-Association with illness: Yes<br>-Heritability: Strong evidence from inherited SCN8A variant and shared neuroimaging traits between affected/unaffected family members<br>-Family Cosegregation: Studies both affected pts and unaffected siblings<br>-State-Independence: Resting-state fMRI independent of seizure activity; abnormalities present in seizure-free unaffected siblings<br>-Higher frequency in unaffected relatives: Siblings > HC	5/5	-Reliable Measurement: Rigorous resting-state fMRI with FCD analysis, proper preprocessing, and validated statistical methods<br>-Association with disease or treatment: FCD alterations are directly linked to SCN8A variant-related epilepsy pathophysiology and seizure generation<br>-Genetic Mediation: Shared patterns in siblings indicate genetic influence	3/3	Novel genetic variant with family validation, sophisticated fMRI analysis
21	Yaakub et al. (2020) mTLE Alpha Network Endophenotypes and relatives [63]	-Association with illness: Demonstrates significant reduction in peak alpha frequency across parietal and occipital electrodes in pts vs. HC	5/5	-Reliable Measurement: Multi-modal approach combining EEG spectral analysis and simultaneous EEG-fMRI with rigorous preprocessing	3/3	Family validation with sophisticated EEG-fMRI analysis

		-Heritability: Strong evidence from shared EEG and fMRI network alterations between affected patients and unaffected relatives<br>-Family Cosegregation: Asymptomatic first-degree relatives also had abnormalities<br>-State-Independence: Alpha abnormalities present in seizure-free unaffected relatives; PAF reductions persist regardless of seizure activity<br>**-**Higher frequency in unaffected relatives: Relatives > HC		-Association with disease or treatment: Alpha abnormalities and sensorimotor network alterations are clearly linked to mTLE pathophysiology<br>-Genetic Mediation: Compelling evidence from family cosegregation showing shared abnormalities in unaffected relatives		
22	Tan et al. (2020) Cognitive Impairments in TLE pts and their siblings [67]	-Association with illness: TLE pts showed significant deficits in the information test, arithmetic test, digit symbol substitution test, block design test, and backward digit span test vs. HC<br>-Heritability: Yes<br>-Family Cosegregation: Shared visuospatial deficits between affected patients and unaffected siblings<br>-State-Independence: Asymptomatic siblings showed cognitive deficits<br>**-**Higher frequency in unaffected relatives: Siblings > HC	5/5	-Reliable Measurement: - Comprehensive neuropsychological battery using standardized tests (WAIS-RC, MMSE)<br>-Association with disease or treatment: Cognitive deficits are consequences of the disease<br>-Genetic Mediation: Shared patterns in siblings indicate genetic influence	3/3	Comprehensive neuropsychological assessment with family validation
23	de Lange et al. (2020) Modifier genes in *SCN1A*-related epilepsy [77]	-Association with illness: Statistical excess of variants in epilepsy genes found in extreme phenotypes<br>-Heritability: Not directly assessed<br>-State-Independence: Genotype present regardless of disease course<br>-Family Cosegregation: Not assessed	2/5	-Reliable Measurement: Whole-exome sequencing<br>-Association with disease or treatment: Clear association with SCN1A-related epilepsy severity<br>-Genetic Mediation: Direct focus on genetic variants as modifiers of phenotype	3/3	Validated genetic study

		-Higher frequency in unaffected relatives: Not assessed				
24	Caciagli et al. (2020) Motor hyperactivation in fMRI in JME patients and their siblings [46]	- Association with illness: Motor hyperactivation in JME pts<br>- Heritability: Supported by its presence in unaffected siblings; family study design<br>-Family Cosegregation: Both patients and siblings show motor hyperactivation<br>-State-Independence: Present regardless of seizure activity, present during cognitive tasks<br>-Higher frequency in unaffected relatives: Siblings > HC	5/5	-Reliable Measurement: - Standardized fMRI protocols and analysis<br>-Association with disease or treatment: Clear association with JME, modulated by seizure activity<br>-Genetic Mediation: ROC analysis shows accurate discrimination of patients and siblings from controls	3/3	Sophisticated fMRI analysis with family validation
25	Long et al. (2020) Hippocampal abnormalities in sporadic TLE and their siblings [50]	- Association with illness: Reduced hippocampal volumes and shape alterations in TLE pts<br>- Heritability: Same abnormalities in unaffected siblings<br>-Family Cosegregation: Strong patient-sibling correlation<br>-State-Independence: Siblings show similar patterns without having seizures<br>-Higher frequency in unaffected relatives: Siblings vs. HC	5/5	-Reliable Measurement: Gold-standard, semi-automated volumetry/shape analysis<br>-Association with disease or treatment: Hippocampal atrophy is a core disease feature in TLE<br>-Genetic Mediation: Shared patterns in siblings indicate genetic influence	3/3	Neuroimaging, volumetric analysis with family validation
26	Gesche (2020) The clinical spectrum of familial and sporadic IGE [85]	-Association with illness: Yes<br>-Heritability: ~27% of cases have a family history<br>-Family Cosegregation: Familial cases identified, and pedigrees analyzed, but clinical features did not differ<br>-State-Independence: Yes<br>-Higher frequency in unaffected relatives: Not assessed	4/5	-Reliable Measurement: Standardized clinical and genetic family history<br>- Association with disease or treatment: Yes<br>-Genetic Mediation: Familial clustering suggests genetic mediation but does not identify new endophenotypes	3/3	Comparison of familial and sporadic IGE

27	Caciagli et al. (2019) Abnormal hippocampal structure and function in JME and their siblings [48]	-Association with illness: Demonstrates significant hippocampal abnormalities in JME vs HC<br>-Heritability: Yes<br>-Family Cosegregation: Unaffected siblings<br>-State-Independence: Structural abnormalities persist regardless of seizure activity<br>-Higher frequency in unaffected relatives: Siblings > HC	5/5	-Reliable Measurement: Standardized fMRI protocols and analysis<br>-Association with disease or treatment: Yes<br>-Genetic Mediation: Unaffected siblings show identical patterns	3/3	Sophisticated fMRI analysis with family validation
28	Yaakub et al. (2019) Temporal Lobe Morphology in mTLE+HS and relatives [53]	-Association with illness: Yes<br>-Heritability: Inferred from shared traits between relatives<br>-Family Cosegregation: First-degree asymptomatic relatives<br>-State-Independence: Yes<br>-Higher frequency in unaffected relatives: Relatives vs. HC	5/5	-Reliable Measurement: Uses validated FreeSurfer processing, ENIGMA consortium pipeline, rigorous quality checks, and Monte Carlo corrections for multiple comparisons<br>-Association with disease or treatment: yes<br>-Genetic Mediation: Yes	3/3	Morphology analysis with family validation
29	Wandschneider et al. (2019) Developmental MRI Markers in JME and Siblings [49]	-Association with illness: Increased curvature and surface area in prefrontal/cingulate cortices in JME pts vs. HC<br>-Heritability: Strong evidence from shared developmental markers between affected pts and unaffected siblings<br>-Family Cosegregation: Strong patient-sibling correlation<br>-State-Independence: Structural developmental markers present regardless of seizure activity; siblings show similar patterns without having seizures	5/5	-Reliable Measurement: Sophisticated surface-based morphometry with multiple complementary metrics<br>-Association with disease or treatment: Morphological anomalies explain cognitive impairments and network dysfunction in JME<br>-Genetic Mediation: Shared patterns in siblings indicate genetic influence	3/3	Sophisticated morphometry study with family validation

		-Higher frequency in unaffected relatives: Relatives vs. HC				
30	Tangwiriyasakul et al. (2019)<br>Functional hypersynchrony in sensorimotor fMRI in GGE and relatives<br>[42]	-Association with illness: Increased sensorimotor blood in pts<br>oxygen level–dependent network synchrony in pts<br>-Heritability: Elevated synchrony found in unaffected first-degree relatives<br>-State-Independence: Trait is present at rest (interictal)<br>-Family Cosegregation: Yes<br>-Higher frequency in unaffected relatives: Relatives > HC	5/5	-Reliable Measurement: Uses simultaneous EEG-fMRI<br>-Association with disease or treatment: Sensorimotor hypersynchrony relevant to seizure susceptibility<br>-Genetic Mediation: Study design excludes environmental/iatrogenic effect; genetic basis likely	3/3	Sophisticated EEG-fMRI study with family validation
31	Schraegle (2017)<br>Paternal anxiety in pediatric epilepsy and their fathers<br>[39]	-Association with illness: Anxiety is clearly associated with pediatric epilepsy (47% with elevated anxiety vs. 15-20% in the general epilepsy population)<br>-Heritability: Parental psychiatric history suggests<br>-State-Independence: Risk is seen in both controlled and intractable epilepsy<br>-Family Cosegregation: Parental psychiatric history suggests<br>-Higher frequency in unaffected relatives: Fathers > HC	5/5	-Reliable Measurement: Uses validated questionnaires<br>-Association with disease or treatment: Anxiety was more severe in youth with intractable epilepsy<br>-Genetic Mediation: Shared patterns in fathers indicate genetic influence	3/3	Psychiatric comorbidity analysis with family validation
32	Wight et al. (2016)<br>Genetic Linkage in JME Subsyndromes<br>[79]	-Association with illness: EEG 3.5-6.0 Hz polyspike waves associated with different JME subsyndromes<br>-Heritability: Partially met - Genetic linkage demonstrated, but focused on chromosomal loci rather than a formal heritability assessment	3/5	-Reliable Measurement: Rigorous video-EEG methodology with standardized protocols and precise genotyping<br>-Association with disease or treatment: EEG polyspike waves are directly linked to seizure generation mechanisms<br>-Genetic Mediation: Linkage analysis identified specific loci	3/3	Extensive multigenerational family genetic linkage study

		-Family Cosegregation: EEG traits cosegregated with disease status across multiple generations in three large pedigrees<br>-State-Independence: EEG polyspike waves present in clinically asymptomatic family members<br>-Higher frequency in unaffected relatives: Partially met - Pedigrees partially support				
33	Carvalho et al. (2016)<br>Cognitive performance in JME<br>[71]	-Association with illness: Shows executive dysfunction differences between JME subgroups<br>-Heritability: Not assessed<br>-Family Cosegregation: Not assessed<br>-State-Independence: Abnormalities present in different JME phenotypes<br>-Higher frequency in unaffected relatives: Not assessed	2/5	-Reliable Measurement: Validated neuropsychological tests<br>-Association with disease or treatment: Clear link between reflex traits and cognitive performance patterns<br>-Genetic Mediation: No direct heritability, family data or genetic analysis	2/3	Neuropsychological study
34	Shen et al. (2015)<br>BDNF Val66Met in TLE<br>[80]	-Association with illness: BDNF Met allele less frequent in TLE and particularly TLE+HS, possibly protective<br>-Heritability: Not assessed - Population-based study<br>-Family Cosegregation: Not assessed<br>-State-Independence: Yes<br>-Higher frequency in unaffected relatives: Not assessed	2/5	-Reliable Measurement: Correct genotyping and imaging, moderate sample for imaging<br>-Association with disease or treatment: BDNF variant is associated with disease risk<br>-Genetic Mediation: Direct (by design: candidate gene)	3/3	Genotype-fenotype assessment study
35	Chowdhury et al. (2015)<br>Motor evoked potential polyphasia in IGE and first-degree relatives	-Association with illness: MEP polyphasia in IGE patients<br>-Heritability: Present in first-degree relatives<br>-Family Cosegregation: Polyphasia present in both patients and relatives	5/5	- Accurate Measurement: Quantitative TMS MEP phase counting<br>- Association with disease or treatment: MEP polyphasia observed in IGE patients and unaffected relatives, independent of treatment.	3/3	Sophisticated TMS, MEP analysis wth family validation

	[64]	-State-Independence: TMS-evoked responses independent of seizure state<br>-Higher frequency in unaffected relatives: Relatives showed significantly more polyphasic MEPs than HC		- Genetic Mediation: Presence in unaffected relatives suggests a genetic basis		
36	Iqbal et al., (2015) Neurocognitive in JME and their siblings [70]	-Association with illness: Executive dysfunction in patients vs. HC<br>-Heritability: Siblings show cognitive deficits<br>-Family Cosegregation: Similar profiles found in affected and siblings<br>-State-Independence: Cognitive deficits occurred independently of EEG activity during video-EEG monitoring; deficits were present regardless of seizure activity<br>-Higher frequency in unaffected relatives: Siblings > HC	5/5	-Accurate Measurement: Comprehensive neuropsychological battery with validated instruments, controlled testing conditions, video-EEG monitoring<br>-Association with disease or treatment: Executive dysfunction relates to JME symptoms<br>-Genetic Mediation: Shared patterns in siblings indicate genetic influence	3/3	Comprehensive neuropsychological assessment with family validation
37	Uchida et al. 2015 Praxis Induction in JME [74]	-Association with illness: PI as a trait in JME, related to worse prognosis<br>- Heritability: Not assessed<br>-Family Cosegregation: Not assessed<br>-State-Independence: Present regardless of seizure state, persistent throughout life<br>-Higher frequency in unaffected relatives: Not assessed	2/5	-Reliable Measurement: Video-EEG confirmed, standardized neuropsychological protocol<br>-Association with disease or treatment: Strong association with worse prognosis in JME - reduced drug response, loss of chronosensitivity<br>-Genetic Mediation: No direct heritability, family data or genetic analysis	2/3	Video-EEG confirmed, neuropsychological protocol
38	Whelan et al. (2015) White matter alterations in TLE and their siblings [55]	-Association with illness: DTI-based WM alterations are present in MRI-negative MTLE pts<br>-Heritability: Asymptomatic siblings had the same abnormalities	5/5	-Reliable Measurement: DTI, tractography/voxelwise methodologies<br>-Association with disease or treatment: White matter alterations are specific to MTLE	3/3	Sophisticated DTI, tractography/voxelwise study with family validation

		-State-Independence: Abnormalities persist in siblings with no seizures<br>-Family Cosegregation: Pts > siblings > HC in abnormalities<br>-Higher frequency in unaffected relatives: Siblings > HC		-Genetic Mediation: Shared patterns in siblings indicate genetic influence		
39	Addis et al. (2014) Migraine in Rolandic Epilepsy [84]	-Association with illness: A Clear association between migraine and Rolandic epilepsy families has been demonstrated through epidemiological analysis<br>-Heritability: Partially met - Strong evidence from linkage analysis and family aggregation, but a formal heritability coefficient was not calculated<br>-Family Cosegregation: Studies showing migraine clustering in RE families beyond seizure effects<br>-State-Independence: Migraine occurs independently of seizure activity<br>-Higher frequency in unaffected relatives: Partially met - mentions previous sibling studies	3/5	-Reliable Measurement: Rigorous genome-wide linkage analysis with appropriate statistical methodology and family-based design<br>-Association with disease or treatment: Migraine phenotype clearly linked to RE pathophysiology and genetic susceptibility<br>-Genetic Mediation: Strong evidence through linkage analysis showing genetic influence on migraine susceptibility in rolandic epilepsy families	3/3	Family-based linkage analysis
40	Chowdhury et al. (2014) Cognitive Endophenotypes in IGE and their relatives [68]	-Association with illness: Demonstrates significant cognitive impairments in IGE pts vs. HC across multiple domains (nonverbal reasoning, verbal fluency, attention, working memory)<br>-Heritability: Strong genetic component in IGE<br>-Family Cosegregation: Cognitive deficits seen in relatives	5/5	-Reliable Measurement: Standardized neuropsychological tests<br>-Association with disease or treatment: Cognitive deficits are clearly linked to IGE pathophysiology and frontal lobe dysfunction<br>-Genetic Mediation: Strong evidence from family cosegregation showing shared cognitive impairments in unaffected relatives	3/3	Neuropsychological assessment with family validation

		-State-Independence: Cognitive deficits present in seizure-free unaffected relatives, independent of seizure activity or medication effects<br>-Higher frequency in unaffected relatives: Relatives > HC				
41	Wandschneider et al. (2014)<br>Motor co-activation in JME and their siblings<br>[47]	-Association with illness: Abnormal motor cortex co-activation with cognitive load<br>-Heritability: Demonstrated in unaffected siblings, suggesting a genetic basis<br>-Family Cosegregation: Siblings show similar abnormalities<br>-State-Independence: Present regardless of seizure activity<br>-Higher frequency in unaffected relatives: Siblings > HC	5/5	-Reliable Measurement: fMRI with standardized working memory task<br>-Association with disease or treatment: Linked to cognitive/motor features in JME-Genetic Mediation: Shared patterns in siblings indicate genetic influence	3/3	Sophisticated fMRI study with family validation
42	Chowdhury et al. (2014)<br>Brain network study in IGE and its relatives<br>[61]	-Association with illness: EEG-derived network abnormalities found in IGE pts vs HC<br>-Heritability: Same network abnormalities found in unaffected first-degree relatives<br>-Family co-segregation: Relatives of affected patients are directly studied and display the marker<br>-State-independence: Present in unaffected relatives (not dependent on seizures or medication).<br>-Higher frequency in unaffected relatives: Relatives > HC	5/5	-Accurate measurement: Quantitative EEG and graph theory, robust group design<br>-Association with disease/treatment: Network metrics robustly separate patients/family from HC<br>-Genetic mediation: Strongly suggested via familial patterns, though no genotyping in this study	3/3	Quantitative EEG study with family validation
43	Alhusaini et al. (2013)<br>Subcortical Volume Heritability in MTLE and their siblings	-Association with illness: MTLE+HS pts showed significant volume deficits in the ipsilateral hippocampus, amygdala, thalamus, and bilateral putamen vs. HC	1/5	-Reliable Measurement: Validated MRI-based volume measurement<br>-Association with disease or treatment: Volume deficits are clearly associated	2/3	MRI-based volume measurement with family study

	[43]	-Heritability: Not met<br>-Family Cosegregation: Not met - Volume deficits were not present in unaffected siblings, except for a non-significant trend in the amygdala<br>-State-Independence: No systematic evaluation of trait persistence across disease states<br>-Higher frequency in unaffected relatives: Siblings had similar results as HC		with MTLE+HS pathophysiology and seizure-related damage<br>-Genetic Mediation: Volume deficits showed no evidence of genetic mediation		
44	Tikka et al. (2013) Quantitative EEG in JME<br>[37]	-Association with illness: Yes<br>-Heritability: Not assessed<br>-State-Independence: Yes<br>-Family Cosegregation: Not assessed<br>-Higher frequency in unaffected relatives: Not assessed	2/5	-Reliable Measurement: Yes<br>-Association with disease or treatment: Yes<br>-Genetic Mediation: No direct heritability, family data or genetic analysis	2/3	Quantitative EEG study
45	Scanlon et al., (2013) Brain Structure Volumes in TLE and their siblings<br>[52]	-Association with illness: Cerebral white matter volume deficits in TLE pts<br>-Heritability: High heritability of brain structure volumes<br>-Family Cosegregation: Unaffected siblings also showed volume deficits<br>- State-Independence: Structural measures independent of seizure state<br>-Higher frequency in unaffected relatives: Siblings > HC	5/5	-Accurate Measurement: MRI-based quantitative measurements<br>-Association with disease or treatment: Deficits associated with TLE<br>-Genetic Mediation: Shared patterns in siblings indicate genetic influence	3/3	MRI-based Quantitative study with family validation
46	Verotti et al. (2013) Neuropsychological impairment in Rolandic Epilepsy pts and their siblings<br>[69]	-Association with illness: Neuropsychological impairments in RE patients<br>-Heritability: Demonstrated in unaffected siblings, suggesting a genetic basis<br>-Family Cosegregation: Impairments present in both patients and siblings	5/5	-Reliable Measurement: Standardized neuropsychological tests<br>-Association with disease or treatment: Clear association with RE and present even before treatment<br>-Genetic Mediation: Shared patterns in siblings indicate genetic influence	3/3	Neuropsychological assessment with family validation

		-State-independence: Present regardless of seizure activity<br>-Higher frequency in unaffected relatives: Siblings > HC				
47	Smith et al. (2012) Neurocognitive endophenotype in Rolandic epilepsy patients and their siblings<br>[89]	-Association with illness: Language and attention deficits demonstrated in pts vs. population means<br>-Heritability: Inferred from shared traits between affected/unaffected siblings<br>-Family Cosegregation: Unaffected siblings show similar neurocognitive impairments<br>-State-Independence: Deficits persist after seizure remission<br>-Higher frequency in unaffected relatives: Siblings vs. population means	5/5	-Reliable Measurement: Standardized neurocognitive battery with established norms<br>-Association with disease or treatment: Language/attention deficits linked to RE pathophysiology<br>-Genetic Mediation: Shared patterns in siblings indicate genetic influence	3/3	Neuropsychological assessment with family validation
48	Clemens et al. (2012) EEG-LORETA Endophenotypes of IGE<br>[65]	-Association with illness: Demonstrates syndrome-specific EEG patterns in JME, absence seizures, EGTCS<br>-Heritability: Not assessed<br>-Family Cosegregation: No family members or unaffected siblings studied; only patient-control comparisons<br>-State-Independence: Partially met - Uses interictal EEG background activity, but doesn't systematically evaluate persistence across disease states<br>-Higher frequency in unaffected relatives: Not assessed	1/5	-Reliable Measurement: Rigorous LORETA source localization<br>-Association with disease or treatment: EEG endophenotypes clearly linked to syndrome-specific seizure propensities<br>-Genetic Mediation: No direct heritability, family data or genetic analysis	2/3	Syndrome-specific EEG pattern evaluation
49	Beniczky et al. (2012) Reflex epileptic traits in JME<br>[75]	-Association with illness: Cognitive task modulation of EDs in JME<br>-Heritability: Not assessed	2/5	-Reliable Measurement: Standardized EEG protocol with statistical control<br>-Association with disease or treatment: Reflex epileptic traits in JME	2/3	Reflex epileptic trait assessment

		-State-Independence: Trait present regardless of current seizure activity, seizure-free and non-seizure-free pts assessed<br>-Family Cosegregation: Not assessed<br>-Higher frequency in unaffected relatives: Not assessed		-Genetic Mediation: No direct heritability, family data or genetic analysis		
50	Guaranha et al. (2011) Reflex/psychiatric traits in JME prognosis [76]	-Association with illness: Reflex/psychiatric traits linked to prognosis in JME<br>-Heritability: No direct heritability estimates<br>-Family Cosegregation: Not assessed<br>-State-Independence: Reflex traits showed parallel evolution with seizure control over time, suggesting they are stable characteristics rather than transient effects<br>-Higher frequency in unaffected relatives: Not assessed	2/5	-Accurate Measurement: Structured video-EEG, psychiatric scales, follow-up for at least three years<br>-Association with disease or treatment: Traits linked to seizure control<br>-Genetic Mediation: No direct heritability, family data or genetic analysis	2/3	Neuropsychiatric and neurophysiologic follow-up study
51	Brazzo et al. (2011) EEG and pattern reversal visual evoked potential assessment in photosensitive epilepsy and IGE [57]	-Association with illness: Demonstrates visual habituation differences between photosensitive pts vs. HC and IGE pts<br>-Heritability: Not assessed<br>-Family Cosegregation: Not assessed<br>-State-Independence: Partially met - Uses cross-sectional design but doesn't systematically evaluate state-independence<br>-Higher frequency in unaffected relatives: Not assessed	1/5	-Reliable Measurement: EEG and pattern reversal visual evoked potential assessment<br>-Association with disease or treatment: Photosensitive patients differ from IGE pts<br>-Genetic Mediation: No direct heritability, family data or genetic analysis	2/3	EEG and pattern reversal visual evoked potential assessment
52	Mula et al. (2010) – Interictal Dysphoric Disorder	-Association with illness: Partially met - Interictal Dysphoric Disorder (IDD) is reported in ~22% of epilepsy patients,	0/5	-Reliable Measurement: Uses validated Interictal Dysphoric Disorder Inventory	1/3	Systematic psychiatric

	[82]	showing significant mood/behavioral symptom burden. However, it overlaps with periictal dysphoric symptoms (PDS), which are tied to seizure timing.<br>-Heritability: Not assessed<br>-Family Cosegregation: No family or unaffected sibling data reported<br>-State-Independence: Not assessed<br>-Higher frequency in unaffected relatives: Not assessed		-Association with disease or treatment: Partially met<br>-Genetic Mediation: No direct heritability, family data or genetic analysis		phenotyping with a large sample size
53	Boutros et al., 2006 Auditory-Evoked Responses and Sensory Gating in Focal Epilepsy [58]	-Association with illness: P200 amplitude reduction and gating deficits found in epilepsy patients<br>-Heritability: Not assessed<br>-Family Cosegregation: Not assessed<br>-State-independence: Not assessed<br>-Higher frequency in unaffected relatives: Not assessed	1/5	-Reliable Measurement: Auditory-Evoked potentials and EEG assessment<br>-Association with disease or treatment: Partially met<br>-Genetic Mediation: No direct heritability, family data or genetic analysis	1/3	Auditory-Evoked responses assessment

Supplementary Table S1. Performance of epilepsy endophenotype studies under different validation criteria

ABBREVIATIONS: BDNF: brain-derived neurotrophic factor, DRE: drug-resistant epilepsy, DTI: diffusion tensor imaging, EEG: electroencephalography, EEM: epilepsy with eyelid myoclonia, FCD: functional connectivity density, FE: focal epilepsy, fMRI: functional magnetic resonance imaging, GABA: gamma-aminobutyric acid, GGE: genetic generalized epilepsy, GTCA: epilepsy with generalized tonic-clonic seizures alone, HC: healthy controls, HS: hippocampal sclerosis, IGE: idiopathic generalized epilepsy, JME: juvenile myoclonic epilepsy, LORETA: low resolution electromagnetic tomography, MEG: magnetoencephalography, MEP: magnetic evoked potential, MMSE: mini-mental state examination, MTLE: mesial temporal lobe epilepsy, pts: patients, PI: praxis induction, pts: patients, qT1 and qT2: quantitative T1 and T2 relaxometry, RE: rolandic epilepsy, ROC: receiver-operating characteristic, TLE: temporal lobe epilepsy, TMS: transcranial magnetic stimulation, vs: versus, VEP: visual evoked potential, vs: versus, WAIS: Wechsler adult intelligence scale
