# Supplementary material for: Endophenotype Research in Epilepsy Across Time
Source: Brain Sci. 2025 Nov 27;15(12):1275. doi: 10.3390/brainsci15121275 (PMC12730710; doi:10.3390/brainsci15121275)
Supplement: Supplementary file 1 [file brainsci-15-01275-s001.zip › Supplementary Table S2-Scoring Codebook.pdf]

Supplementary Table S2. Operational Scoring Codebook for Endophenotype Validation Frameworks

TRADITIONAL FRAMEWORK (Gottesman & Gould, 2003)

-Criterion 1: Association with Illness

DEFINITION: A Clear relationship between endophenotype and epilepsy diagnosis

SCORING CRITERIA:

✓ MET (1 point):	✗ NOT MET (0 points):
• Statistically significant difference between patients and controls ($p < 0.05$)<br>• OR association in case-control studies with a non-overlapping 95% CI	• No statistical comparison provided<br>• Non-significant results ($p \geq 0.05$)<br>• Descriptive data only without formal testing

EXAMPLES:

✓ Example 1: Patients with epilepsy had a significantly increased IQR of the number of magnetic evoked potential phases compared with controls. [64]

✗ Example 2: Interictal Dysphoric Disorder is reported in ~22% of epilepsy patients, showing significant mood/behavioral symptom burden. However, it overlaps with periictal dysphoric symptoms, which are tied to seizure timing. [82]

-Criterion 2: Heritability

DEFINITION: Evidence of genetic contribution to trait variance

SCORING CRITERIA:

✓ MET (1 point):	✗ NOT MET (0 points):
• Formal heritability estimate<br>• Siblings study<br>• Quantitative genetic analysis<br>• Family aggregation with statistical testing	• No genetic data provided<br>• Descriptive family patterns without statistical analysis<br>• Citation of heritability from other studies without original data

EXAMPLES:

✓ Example 1: Strong evidence from shared electroencephalography (EEG) and functional magnetic resonance (fMRI) network alterations between affected patients and unaffected relatives. [63]

✗ Example 2: Studies on juvenile myoclonic epilepsy (JME) patients and their healthy siblings showed that JME is associated with subtle cognitive deficits. [73]

-Criterion 3: Family Co-segregation

DEFINITION: Trait presence in unaffected family members of patients

SCORING CRITERIA:

✓ MET (1 point):	✗ NOT MET (0 points):
• Inclusion of unaffected first-degree relatives (siblings, parents, offspring)<br>• Statistical comparison showing trait present in relatives at intermediate levels<br>• Family-based design with ≥3 groups (patients, relatives, unrelated controls)	• No family member data<br>• Only affected family members included<br>• Relatives not assessed for the endophenotype

EXAMPLES:

✓ Example 1: Both patients and siblings show motor hyperactivation in fMRI. [46]

✗ Example 2: Volume deficits were not present in unaffected siblings, except for a non-significant trend in the amygdala. [43]

-Criterion 4: State Independence

DEFINITION: Trait persistence regardless of acute illness episodes or treatment status

SCORING CRITERIA:

✓ MET (1 point):	✗ NOT MET (0 points):
• Trait present during remission or interictal periods<br>• No correlation with recent seizure frequency or medication changes<br>• Present before illness onset (prodromal/at-risk populations)	• Only assessed during the acute/active disease phase<br>• Varies with seizure frequency or treatment<br>• No assessment of temporal stability<br>• State-dependent by definition (e.g., ictal markers)

EXAMPLES:

✓ Example 1: Structural imaging measures (cortical thickness, subcortical volumes) persist independent of seizure activity. [45]

✗ Example 2: EEG abnormalities were assessed only during active seizure periods.

-Criterion 5: Higher Frequency in Unaffected Relatives

DEFINITION: Intermediate trait expression in relatives compared to the general population

SCORING CRITERIA:

✓ MET (1 point):	✗ NOT MET (0 points):
• Quantitative trait shows gradient: patients > relatives > controls<br>• Categorical trait shows intermediate prevalence in relatives<br>• Statistical testing confirms relatives differ from both patients and controls	• Relatives indistinguishable from controls<br>• Relatives identical to patients (no gradient)<br>• No comparison between relatives and controls<br>• Not assessed, only mentioned previous sibling studies

EXAMPLES:

✓ Example 1: Juvenile absence epilepsy (JAE) siblings had an intermediate position between patients and controls for attention/psychomotor speed and global executive function, with no statistically significant differences against either group. [66]

✗ Example 2: No relatives were assessed

ENDOPHENOTYPE 2.0 FRAMEWORK (Liu & Gershon, 2024)

<u>Criterion 1: Reliable Measurement</u>

DEFINITION: Validated, standardized, reproducible assessment methods with established psychometric properties

SCORING CRITERIA:

✓ MET (1 point):	✗ NOT MET (0 points):
• Standardized assessment protocol described<br>• Validated neuroimaging protocol with quality control<br>• Established genetic/molecular assay with replication	• Novel measure without validation<br>• No reliability data provided<br>• Inconsistent assessment methods across sites

EXAMPLES:

✓ Example 1: Uses validated FreeSurfer processing, ENIGMA consortium pipeline, rigorous quality checks, and Monte Carlo corrections for multiple comparisons [53].

✗ Example 2: A Novel EEG connectivity metric was developed for this study without external validation.

Criterion 2: Association with Disease/Treatment

DEFINITION: Clear relationship with clinical outcomes, disease severity, or therapeutic

Response

SCORING CRITERIA:

✓ MET (1 point):	✗ NOT MET (0 points):
• Association with diagnosis (as in Traditional Criterion 1)<br>• Correlation with disease severity/progression<br>• Prediction of treatment response<br>• Association with clinical subtypes/outcomes	• No clinical associations demonstrated<br>• Non-significant results<br>• Purely genetic association without a clinical phenotype link

EXAMPLES:

✓ Example 1: Clinical subtypes clearly associated with seizure outcomes and neuropsychiatric comorbidities [83]

✓ Example 2: GABRG2 Mutations correspond with phenotype spectrum and severity [78]

✗ Example 3: P200 amplitude reduction and gating deficits found in epilepsy patients [58]

Criterion 3: Genetic Mediation

DEFINITION: Evidence of genetic influence through molecular studies, family patterns,

or heritability analysis

SCORING CRITERIA:

✓ MET (1 point):	✗ NOT MET (0 points):
• Molecular genetic findings (specific genes, variants, polygenic scores)<br>• Family co-segregation data (relatives affected)<br>• Formal heritability estimates<br>• Genetic association studies (GWAS, candidate gene)<br>• Twin or adoption studies	• No genetic data presented<br>• Pure population/environmental associations<br>•Theoretical genetic basis without empirical data

• At least ONE of the above with supporting statistical evidence	

FLEXIBILITY NOTE: This criterion is MORE PERMISSIVE than the Traditional Framework!

- Accepts modern genomic approaches (GWAS, PRS) without requiring family data

- Allows population-based genetic associations

- Does not require demonstration in unaffected relatives

- Can be satisfied through molecular genetics alone

EXAMPLES:

✓ Example 1: GABRG2 Loss- and Gain-of-Function Variants - Specific genetic variants identified [78]

✓ Example 2: Modifier genes in SCN1A-related epilepsy syndromes - Direct molecular genetic evidence [77]

✓ Example 3: Motor co-activation in siblings - Trait present in unaffected siblings [47]

✗ Example 5: MRI abnormalities correlated with environmental exposures; no genetic analysis performed.

ADJUDICATION RULES

---

1. AMBIGUOUS CASES:

  - If data are present but statistical significance is not reported: NOT MET

  - If effect size borderline (d = 0.25–0.30): Discuss with senior authors

  - If indirect evidence cited from literature: NOT MET (original data required)

2. PARTIAL EVIDENCE:

- "Trending" results (p = 0.05–0.10): NOT MET
- Subgroup analyses significant but overall analysis not: NOT MET
- Post-hoc exploratory findings: Accept if controlled adequately for multiple testing

3. STUDY DESIGN CONSIDERATIONS:

- Pilot studies (n < 20): Accept if methodology sounds, note in quality assessment
- Multisite studies: Require demonstration of cross-site reliability
- Secondary analyses: Accept if endophenotype assessment was primary/co-primary aim

4. FRAMEWORK-SPECIFIC RULES:

Traditional Framework:

- All 5 criteria are independent; must meet each separately
- Family data required for Criteria 2, 3, 5
- State-independence (Criterion 4) strictly enforced

Endophenotype 2.0:

- Criterion 3 can be satisfied in multiple ways (more flexible)
- State-dependent markers are acceptable if they meet other criteria
- Population-based genetic associations sufficient for Criterion 3
