# Supplementary material for: Endophenotype Research in Epilepsy Across Time
Source: Brain Sci. 2025 Nov 27;15(12):1275. doi: 10.3390/brainsci15121275 (PMC12730710; doi:10.3390/brainsci15121275)
Supplement: Supplementary file 1 [file brainsci-15-01275-s001.zip › Supplementary Table S3-Codes.pdf]

Bibliometrix analysis code

```
library(bibliometrix)

S = convert2df("scopus.bib",dbsource = "scopus",format = "bibtex")

W = convert2df("wos.bib",dbsource = "isi", format = "bibtex")

Database = mergeDbSources(S,W, remove.duplicated = TRUE)

dim(Database)

library(openxlsx)

write.xlsx(Database, file = "database.xlsx")

biblioshiny()
```

INTER-RATER AGREEMENT ANALYSIS

```
library(irr)
```

Traditional criteria

```
# Rater data
rater1 <- c(2, 2, 4, 2, 2, 2, 2, 5, 1, 2, 5, 2, 3, 1, 2, 5, 5, 2, 4, 5, 5, 5, 2, 3, 5, 4, 5, 5, 2, 5, 5, 5, 4,
5, 5, 5, 5, 5, 3, 3, 2, 5, 5, 2, 3, 3, 5, 5, 5, 1, 2, 0, 1)
rater2 <- c(1, 2, 4, 2, 1, 2, 3, 2, 5, 3, 5, 1, 3, 2, 2, 5, 5, 1, 4, 5, 5, 5, 2, 3, 5, 4, 5, 5, 2, 5, 5, 5, 4,
5, 5, 5, 5, 5, 5, 2, 2, 5, 5, 1, 5, 4, 5, 5, 5, 2, 2, 1, 2)

df <- data.frame(rater1, rater2)

interpret_kappa <- function(kappa_value) {
  if (kappa_value < 0) return("Poor")
  if (kappa_value <= 0.20) return("Slight")
  if (kappa_value <= 0.40) return("Fair")
  if (kappa_value <= 0.60) return("Moderate")
  if (kappa_value <= 0.80) return("Substantial")
  return("Almost Perfect")
}

cat("Number of ratings:", length(rater1), "\n")
cat("Rater 1 range:", min(rater1), "-", max(rater1), "\n")
cat("Rater 2 range:", min(rater2), "-", max(rater2), "\n\n")
```

```r
# UNWEIGHTED Cohen's Kappa
kappa_unweighted <- kappa2(df, weight = "unweighted")
cat("=== UNWEIGHTED KAPPA (Nominal) ===\n")
print(kappa_unweighted)
cat("\nCohen's Kappa:", round(kappa_unweighted$value, 3), "\n")
cat("P-value:", format.pval(kappa_unweighted$p.value, digits = 3), "\n")
cat("Agreement level:", interpret_kappa(kappa_unweighted$value), "\n\n")

# LINEAR WEIGHTED Kappa
kappa_linear <- kappa2(df, weight = "equal")
cat("=== WEIGHTED KAPPA – Linear (Ordinal) ===\n")
print(kappa_linear)
cat("\nLinear Weighted Kappa:", round(kappa_linear$value, 3), "\n")
cat("P-value:", format.pval(kappa_linear$p.value, digits = 3), "\n")
cat("Agreement level:", interpret_kappa(kappa_linear$value), "\n\n")

# QUADRATIC WEIGHTED Kappa
kappa_quadratic <- kappa2(df, weight = "squared")
cat("=== WEIGHTED KAPPA – Quadratic (Ordinal) ===\n")
print(kappa_quadratic)
cat("\nQuadratic Weighted Kappa:", round(kappa_quadratic$value, 3), "\n")
cat("P-value:", format.pval(kappa_quadratic$p.value, digits = 3), "\n")
cat("Agreement level:", interpret_kappa(kappa_quadratic$value), "\n\n")

# CONFUSION MATRIX
confusion_matrix <- table(Rater1 = rater1, Rater2 = rater2)
cat("=== CONFUSION MATRIX ===\n")
print(confusion_matrix)
cat("\n")

# Percent agreement
agreements <- sum(rater1 == rater2)
total <- length(rater1)
percent_agreement <- (agreements / total) * 100

cat("Simple Percent Agreement:", round(percent_agreement, 2), "%\n")
cat("Number of exact agreements:", agreements, "out of", total, "\n\n")

# Save one final kappa output for later use
kappa_result <- kappa_quadratic
```

```
cat("Agreement level (final kappa_result):", interpret_kappa(kappa_result$value), "\n")
```

Endophenotype 2.0

```
# Rater data
rater1 <- c(2, 2, 3, 2, 2, 2, 3, 2, 3, 2, 3, 3, 3, 2, 2, 2, 3, 1, 3, 3, 3, 3, 3, 3, 3, 3, 3, 3, 3, 3, 3, 3, 2,
3, 3, 3, 2, 3, 3, 3, 3, 3, 2, 2, 3, 3, 3, 2, 2, 2, 2, 1, 1)
rater2 <- c(2, 2, 3, 2, 2, 2, 3, 3, 3, 3, 2, 3, 3, 2, 3, 2, 3, 2, 3, 3, 3, 3, 3, 3, 3, 3, 3, 3, 3, 3, 3, 3, 2,
3, 3, 2, 2, 3, 3, 3, 3, 3, 2, 2, 3, 3, 3, 2, 2, 2, 2, 2, 2)

df <- data.frame(rater1, rater2)

interpret_kappa <- function(kappa_value) {
  if (kappa_value < 0) return("Poor")
  if (kappa_value <= 0.20) return("Slight")
  if (kappa_value <= 0.40) return("Fair")
  if (kappa_value <= 0.60) return("Moderate")
  if (kappa_value <= 0.80) return("Substantial")
  return("Almost Perfect")
}

cat("Number of ratings:", length(rater1), "\n")
cat("Rater 1 range:", min(rater1), "-", max(rater1), "\n")
cat("Rater 2 range:", min(rater2), "-", max(rater2), "\n\n")

# UNWEIGHTED Cohen's Kappa
kappa_unweighted <- kappa2(df, weight = "unweighted")
cat("=== UNWEIGHTED KAPPA (Nominal) ===\n")
print(kappa_unweighted)
cat("\nCohen's Kappa:", round(kappa_unweighted$value, 3), "\n")
cat("P-value:", format.pval(kappa_unweighted$p.value, digits = 3), "\n")
cat("Agreement level:", interpret_kappa(kappa_unweighted$value), "\n\n")

# LINEAR WEIGHTED Kappa
kappa_linear <- kappa2(df, weight = "equal")
cat("=== WEIGHTED KAPPA – Linear (Ordinal) ===\n")
print(kappa_linear)
cat("\nLinear Weighted Kappa:", round(kappa_linear$value, 3), "\n")
cat("P-value:", format.pval(kappa_linear$p.value, digits = 3), "\n")
cat("Agreement level:", interpret_kappa(kappa_linear$value), "\n\n")
```

```
# QUADRATIC WEIGHTED Kappa
kappa_quadratic <- kappa2(df, weight = "squared")
cat("=== WEIGHTED KAPPA – Quadratic (Ordinal) ===\n")
print(kappa_quadratic)
cat("\nQuadratic Weighted Kappa:", round(kappa_quadratic$value, 3), "\n")
cat("P-value:", format.pval(kappa_quadratic$p.value, digits = 3), "\n")
cat("Agreement level:", interpret_kappa(kappa_quadratic$value), "\n\n")

# CONFUSION MATRIX
confusion_matrix <- table(Rater1 = rater1, Rater2 = rater2)
cat("=== CONFUSION MATRIX ===\n")
print(confusion_matrix)
cat("\n")

# Percent agreement
agreements <- sum(rater1 == rater2)
total <- length(rater1)
percent_agreement <- (agreements / total) * 100

cat("Simple Percent Agreement:", round(percent_agreement, 2), "%\n")
cat("Number of exact agreements:", agreements, "out of", total, "\n\n")

kappa_result <- kappa_quadratic

cat("Agreement level (final kappa_result):", interpret_kappa(kappa_result$value), "\n")
```

RANDOM-EFFECTS META-ANALYSIS OF VALIDATION PROPORTIONS AND META-REGRESSION

Analyzing the proportion achieving a perfect score (Score = 3)

```
library(readxl)
library(meta)
library(metafor)
library(dplyr)
library(ggplot2)

data <- read_excel("EN.xlsx")
```

```r
colnames(data) <- c("Study", "Sample_size", "Design", "Modality", "Score")

data$Year <- as.numeric(sub(".*?(\\d{4}).*", "\\1", data$Study))

data$Family_based <- ifelse(data$Design == "Family based", 1, 0)

data$Perfect_score <- ifelse(data$Score == 3, 1, 0)

# For proportion meta-analysis: number achieving perfect score
# Assuming all studies either got perfect score (all n) or didn't (0)
data$n_perfect <- ifelse(data$Perfect_score == 1, data$Sample_size, 0)
data$n_not_perfect <- ifelse(data$Perfect_score == 0, data$Sample_size, 0)

cat("=== DATA OVERVIEW ===\n")
print(head(data, 10))
cat("\nTotal number of studies:", nrow(data), "\n\n")

# Summary statistics
cat("=== DESCRIPTIVE STATISTICS ===\n\n")

cat("Score distribution:\n")
print(table(data$Score))
cat("\nPerfect scores (Score = 3):", sum(data$Perfect_score),
    "out of", nrow(data),
    sprintf("(%.1f%%)", 100*mean(data$Perfect_score)), "\n\n")

cat("Design distribution:\n")
print(table(data$Design))

cat("\nModality distribution:\n")
print(table(data$Modality))

cat("\nPerfect scores by Design:\n")
print(table(data$Design, data$Perfect_score))
cat("Proportion with perfect score:\n")
print(prop.table(table(data$Design, data$Perfect_score), 1))

cat("\nPerfect scores by Modality:\n")
print(table(data$Modality, data$Perfect_score))

cat("\nSample size by design:\n")
```

```
print(aggregate(Sample_size ~ Design, data = data,
          FUN = function(x) c(Mean = round(mean(x), 1),
                              Median = median(x),
                              Min = min(x), Max = max(x))))

cat("\nYear range:", min(data$Year), "-", max(data$Year), "\n\n")
```

Logistic meta-analysis for binary outcome (Perfect Score vs Not)

```
cat("=== LOGISTIC REGRESSION META-ANALYSIS ===\n")
cat("Outcome: Achieving Perfect Score (3 out of 3)\n\n")

# Calculate log odds and variance for each study
# Using correction for zero cells (adding 0.5)
data$events <- data$n_perfect + 0.5
data$n_total <- data$Sample_size + 1
data$non_events <- data$n_total - data$events

data$log_odds <- log(data$events / data$non_events)
data$se_log_odds <- sqrt(1/data$events + 1/data$non_events)
data$var_log_odds <- data$se_log_odds^2

# Random-effects meta-analysis using metafor
cat("--- Overall Random-Effects Model ---\n")
re_model <- rma(yi = log_odds,
          vi = var_log_odds,
          data = data,
          method = "REML",
          slab = Study)
print(re_model)

# Convert to odds ratio
or_overall <- exp(coef(re_model))
or_ci_lower <- exp(coef(re_model) - 1.96 * re_model$se)
or_ci_upper <- exp(coef(re_model) + 1.96 * re_model$se)

cat("\n*** OVERALL ODDS OF ACHIEVING PERFECT SCORE ***\n")
cat("Pooled OR:", round(or_overall, 3), "\n")
cat("95% CI: [", round(or_ci_lower, 3), ",", round(or_ci_upper, 3), "]\n\n")

# Heterogeneity
```

```
cat("=== HETEROGENEITY ===\n")
cat("I² (%):", round(re_model$I2, 2), "\n")
cat("τ² (tau-squared):", round(re_model$tau2, 4), "\n")
cat("H²:", round(re_model$H2, 2), "\n")
cat("Q statistic:", round(re_model$QE, 2), "\n")
cat("p-value for Q:", format.pval(re_model$QEp, digits = 3), "\n")

interpret_i2 <- function(i2) {
  if (i2 < 25) return("Low heterogeneity")
  if (i2 < 50) return("Moderate heterogeneity")
  if (i2 < 75) return("Substantial heterogeneity")
  return("Considerable heterogeneity")
}
cat("Interpretation:", interpret_i2(re_model$I2), "\n\n")
```

META-REGRESSION ANALYSIS

```
cat("=== META-REGRESSION ANALYSIS ===\n\n")

# 1. Study Year
cat("--- 1. STUDY YEAR ---\n")
mr_year <- rma(yi = log_odds, vi = var_log_odds,
           mods = ~ Year, data = data, method = "REML")
print(mr_year)
cat("\nCoefficient interpretation: OR change per year =",
  round(exp(coef(mr_year)[2]), 3), "\n")
cat("95% CI: [", round(exp(coef(mr_year)[2] - 1.96*mr_year$se[2]), 3), ",",
  round(exp(coef(mr_year)[2] + 1.96*mr_year$se[2]), 3), "]\n\n")

# 2. Sample Size (log-transformed for better model fit)
cat("--- 2. SAMPLE SIZE (log-transformed) ---\n")
data$log_sample_size <- log(data$Sample_size)
mr_sample <- rma(yi = log_odds, vi = var_log_odds,
             mods = ~ log_sample_size, data = data, method = "REML")
print(mr_sample)
cat("\n")

# 3. Design (Family-based vs Population-based) - PRIMARY ANALYSIS
cat("--- 3. DESIGN (Family-based vs Population-based) ---\n")
mr_design <- rma(yi = log_odds, vi = var_log_odds,
             mods = ~ Family_based, data = data, method = "REML")
```

```
print(mr_design)

# Calculate odds ratio for family-based design
or_family <- exp(coef(mr_design)[2])
or_ci_lower_family <- exp(coef(mr_design)[2] - 1.96 * mr_design$se[2])
or_ci_upper_family <- exp(coef(mr_design)[2] + 1.96 * mr_design$se[2])

cat("\n*** ODDS RATIO FOR FAMILY-BASED vs POPULATION-BASED ***\n")
cat("OR =", round(or_family, 3), "\n")
cat("95% CI: [", round(or_ci_lower_family, 3), ",",
  round(or_ci_upper_family, 3), "]\n")
cat("p-value:", format.pval(mr_design$pval[2], digits = 3), "\n")
cat("\nInterpretation: Family-based studies have", round(or_family, 2),
  "times the odds of achieving perfect score compared to population-based studies.\n\n")

# 4. Modality
cat("--- 4. MODALITY ---\n")
data$Modality_factor <- relevel(factor(data$Modality), ref = "Clinical")
mr_modality <- rma(yi = log_odds, vi = var_log_odds,
             mods = ~ Modality_factor, data = data, method = "REML")
print(mr_modality)
cat("\n")

# 5. Multivariate meta-regression
cat("--- 5. MULTIVARIATE META-REGRESSION ---\n")
mr_multi <- rma(yi = log_odds, vi = var_log_odds,
          mods = ~ Year + log_sample_size + Family_based + Modality_factor,
          data = data, method = "REML")
print(mr_multi)

# Extract adjusted OR for family-based design from multivariate model
or_family_adj <- exp(coef(mr_multi)["Family_based"])
or_ci_lower_adj <- exp(coef(mr_multi)["Family_based"] -
                1.96 * mr_multi$se["Family_based"])
or_ci_upper_adj <- exp(coef(mr_multi)["Family_based"] +
                1.96 * mr_multi$se["Family_based"])

cat("\n*** ADJUSTED OR FOR FAMILY-BASED DESIGN ***\n")
cat("(Adjusted for year, sample size, and modality)\n")
cat("OR =", round(or_family_adj, 3), "\n")
cat("95% CI: [", round(or_ci_lower_adj, 3), ",",
  round(or_ci_upper_adj, 3), "]\n")
```

```r
cat("p-value:", format.pval(mr_multi$pval["Family_based"], digits = 3), "\n\n")
```

SUBGROUP ANALYSIS BY DESIGN

```r
cat("=== SUBGROUP ANALYSIS: DESIGN ===\n\n")

# Family-based studies
data_family <- data[data$Design == "Family based", ]
re_family <- rma(yi = log_odds, vi = var_log_odds,
                 data = data_family, method = "REML")

cat("FAMILY-BASED STUDIES (n =", nrow(data_family), "):\n")
cat("  Studies with perfect score:", sum(data_family$Perfect_score),
    sprintf("(%.1f%%)", 100*mean(data_family$Perfect_score)), "\n")
cat("  Pooled log-odds:", round(coef(re_family), 3), "\n")
cat("  Pooled OR:", round(exp(coef(re_family)), 3), "\n")
cat("  95% CI: [", round(exp(coef(re_family) - 1.96*re_family$se), 3), ",",
    round(exp(coef(re_family) + 1.96*re_family$se), 3), "]\n")
cat("  I²:", round(re_family$I2, 2), "%\n")
cat("  τ²:", round(re_family$tau2, 4), "\n\n")

# Population-based studies
data_pop <- data[data$Design == "Population based", ]
re_pop <- rma(yi = log_odds, vi = var_log_odds,
              data = data_pop, method = "REML")

cat("POPULATION-BASED STUDIES (n =", nrow(data_pop), "):\n")
cat("  Studies with perfect score:", sum(data_pop$Perfect_score),
    sprintf("(%.1f%%)", 100*mean(data_pop$Perfect_score)), "\n")
cat("  Pooled log-odds:", round(coef(re_pop), 3), "\n")
cat("  Pooled OR:", round(exp(coef(re_pop)), 3), "\n")
cat("  95% CI: [", round(exp(coef(re_pop) - 1.96*re_pop$se), 3), ",",
    round(exp(coef(re_pop) + 1.96*re_pop$se), 3), "]\n")
cat("  I²:", round(re_pop$I2, 2), "%\n")
cat("  τ²:", round(re_pop$tau2, 4), "\n\n")
```

SUBGROUP ANALYSIS BY MODALITY

```r
cat("=== SUBGROUP ANALYSIS: MODALITY ===\n\n")
modality_results <- data.frame()

for (mod in unique(data$Modality)) {
```

```
  data_mod <- data[data$Modality == mod, ]
  n_studies <- nrow(data_mod)
  n_perfect <- sum(data_mod$Perfect_score)
  prop_perfect <- mean(data_mod$Perfect_score)

  cat(mod, "(n =", n_studies, "):\n")
  cat("  Perfect scores:", n_perfect, sprintf("(%.1f%%)", 100*prop_perfect), "\n")

  if (n_studies >= 3) {
    re_mod <- rma(yi = log_odds, vi = var_log_odds,
                  data = data_mod, method = "REML")
    cat("  Pooled OR:", round(exp(coef(re_mod)), 3), "\n")
    cat("  I²:", round(re_mod$I2, 2), "%\n\n")
  } else {
    cat("  (Too few studies for meta-analysis)\n\n")
  }
}
```

SENSITIVITY ANALYSIS

```
cat("=== SENSITIVITY ANALYSIS ===\n\n")

# Leave-one-out analysis
cat("Leave-One-Out Analysis:\n")
loo <- leave1out(re_model)
print(loo, digits = 3)

# Identify influential studies
png("influence_plot.png", width = 12, height = 10, units = "in", res = 300)
inf <- influence(re_model)
plot(inf)
dev.off()
cat("\nInfluence diagnostics plot saved as 'influence_plot.png'\n\n")

# Studies with extreme influence
cat("Studies with Cook's distance > 4/n:\n")
influential_threshold <- 4/nrow(data)
influential_studies <- which(inf$inf$cook.d > influential_threshold)
if (length(influential_studies) > 0) {
  print(data[influential_studies, c("Study", "Score", "Sample_size", "Design")])
} else {
```

```
  cat("No highly influential studies detected.\n")
}
cat("\n")
```

ADDITIONAL: COMPREHENSIVE SUBGROUP ANALYSIS BY DESIGN

```
cat("\n\n")
cat("=======================================================================
=\n")
cat("       COMPREHENSIVE SUBGROUP ANALYSIS BY DESIGN TYPE\n")
cat("=======================================================================
=\n\n")

# Separate datasets
data_family <- data[data$Design == "Family based", ]
data_pop <- data[data$Design == "Population based", ]

cat("DATASET SUMMARY:\n")
cat("Family-based studies: n =", nrow(data_family), "\n")
cat("Population-based studies: n =", nrow(data_pop), "\n\n")

# Run separate meta-analyses
cat("========================================\n")
cat("FAMILY-BASED STUDIES - DETAILED ANALYSIS\n")
cat("========================================\n\n")
re_family <- rma(yi = log_odds, vi = var_log_odds,
               data = data_family, method = "REML", slab = Study)
print(re_family)

or_family_pooled <- exp(coef(re_family))
or_family_lower <- exp(coef(re_family) - 1.96 * re_family$se)
or_family_upper <- exp(coef(re_family) + 1.96 * re_family$se)

cat("\n*** FAMILY-BASED POOLED RESULTS ***\n")
cat("Number of studies:", nrow(data_family), "\n")
cat("Studies with perfect score:", sum(data_family$Perfect_score),
    sprintf("(%.1f%%)\n", 100*mean(data_family$Perfect_score)))
cat("Pooled OR:", round(or_family_pooled, 3), "\n")
cat("95% CI: [", round(or_family_lower, 3), ",", round(or_family_upper, 3), "]\n")
cat("I²:", round(re_family$I2, 2), "%\n")
cat("τ²:", round(re_family$tau2, 4), "\n")
cat("Q statistic:", round(re_family$QE, 2), "\n")
```

```r
cat("p-value for heterogeneity:", format.pval(re_family$QEp, digits = 3), "\n\n")

cat("==================================================\n")
cat("POPULATION-BASED STUDIES - DETAILED ANALYSIS\n")
cat("==================================================\n\n")
re_pop <- rma(yi = log_odds, vi = var_log_odds,
             data = data_pop, method = "REML", slab = Study)
print(re_pop)

or_pop_pooled <- exp(coef(re_pop))
or_pop_lower <- exp(coef(re_pop) - 1.96 * re_pop$se)
or_pop_upper <- exp(coef(re_pop) + 1.96 * re_pop$se)

cat("\n*** POPULATION-BASED POOLED RESULTS ***\n")
cat("Number of studies:", nrow(data_pop), "\n")
cat("Studies with perfect score:", sum(data_pop$Perfect_score),
    sprintf("(%.1f%%)\n", 100*mean(data_pop$Perfect_score)))
cat("Pooled OR:", round(or_pop_pooled, 3), "\n")
cat("95% CI: [", round(or_pop_lower, 3), ",", round(or_pop_upper, 3), "]\n")
cat("I²:", round(re_pop$I2, 2), "%\n")
cat("τ²:", round(re_pop$tau2, 4), "\n")
cat("Q statistic:", round(re_pop$QE, 2), "\n")
cat("p-value for heterogeneity:", format.pval(re_pop$QEp, digits = 3), "\n\n")

# Test for subgroup differences
cat("=============================================\n")
cat("TEST FOR SUBGROUP DIFFERENCES\n")
cat("=============================================\n\n")
subgroup_test <- rma(yi = log_odds, vi = var_log_odds,
                    mods = ~ Design, data = data, method = "REML")
print(subgroup_test)

cat("\n*** BETWEEN-SUBGROUP COMPARISON ***\n")
cat("Difference in log-odds:", round(coef(subgroup_test)[2], 3), "\n")
cat("OR for Family-based vs Population-based:",
    round(exp(coef(subgroup_test)[2]), 3), "\n")
cat("95% CI: [",
    round(exp(coef(subgroup_test)[2] - 1.96*subgroup_test$se[2]), 3), ",",
    round(exp(coef(subgroup_test)[2] + 1.96*subgroup_test$se[2]), 3), "]\n")
cat("p-value:", format.pval(subgroup_test$pval[2], digits = 3), "\n")
cat("QM (test for moderators):", round(subgroup_test$QM, 2), "\n")
cat("p-value for QM:", format.pval(subgroup_test$QMp, digits = 3), "\n\n")
```

SUBGROUP FOREST PLOTS

```
cat("Creating subgroup-specific forest plots...\n\n")

# Forest plot for Family-based studies only
png("forest_family_only.png", width = 20, height = 16, units = "in", res = 300)

# Define extra columns for family-based studies
extra_info_family <- cbind(data_family$Sample_size, data_family$Modality)

forest(re_family,
       xlim = c(-14, 8),
       at = log(c(0.001, 0.01, 0.1, 1, 10, 100)),
       atransf = exp,
       xlab = "Odds Ratio (log scale)",
       header = "Study",
       order = data_family$Year,
       annotate = TRUE,
       addfit = TRUE,
       showweights = FALSE,
       col = "darkblue",
       digits = 2L,
       cex = 0.9,  # Font size
       ilab = extra_info_family,
       ilab.xpos = c(-10.5, -8.5),
       ilab.pos = 4
)

# Manually add column headers
text(c(-10.5, -8.5), re_family$k + 2,
     labels = c("Sample size", "Modality"),
     font = 2, cex = 0.9)

# Add title for subgroup
text(-14, re_family$k + 2.5, pos = 4, cex = 1.1, font = 2, col = "darkblue",
     "FAMILY-BASED STUDIES")

# Model summary at bottom
text(-12, -1, pos = 4, cex = 0.9,
     bquote(paste("Family-based RE Model: OR = ", .(round(or_family_pooled, 2)),
              " [", .(round(or_family_lower, 2)), ", ", .(round(or_family_upper, 2)), "]",
```

```
        ", I"^2, " = ", .(formatC(re_family$I2, digits = 1, format = "f")),
        "%, ", tau^2, " = ", .(formatC(re_family$tau2, digits = 3, format = "f")))))

dev.off()
cat("Family-based forest plot saved as 'forest_family_only.png'\n")

# Forest plot for Population-based studies only
png("forest_population_only.png", width = 20, height = 16, units = "in", res = 300)

# Define extra columns for population-based studies
extra_info_pop <- cbind(data_pop$Sample_size, data_pop$Modality)

forest(re_pop,
    xlim = c(-13, 8),
    at = log(c(0.001, 0.01, 0.1, 1, 10, 100)),
    atransf = exp,
    xlab = "Odds Ratio (log scale)",
    header = "Study",
    order = data_pop$Year,
    annotate = TRUE,
    addfit = TRUE,
    showweights = FALSE,
    col = "darkred",
    digits = 2L,
    cex = 0.9,
    ilab = extra_info_pop,
    ilab.xpos = c(-10.5, -8.5),
    ilab.pos = 4
)

# Manually add column headers
text(c(-10.5, -8.5), re_pop$k + 2,
   labels = c("Sample size", "Modality"),
   font = 2, cex = 0.9)

# Add title for subgroup
text(-12, re_pop$k + 2.5, pos = 4, cex = 1.1, font = 2, col = "darkred",
   "POPULATION-BASED STUDIES")

# Model summary at bottom
text(-11, -1, pos = 4, cex = 0.9,
   bquote(paste("Population-based RE Model: OR = ", .(round(or_pop_pooled, 2)),
```

```
        " [", .(round(or_pop_lower, 2)), ", ", .(round(or_pop_upper, 2)), "]",
        ", I"^2, " = ", .(formatC(re_pop$I2, digits = 1, format = "f")),
        "%, ", tau^2, " = ", .(formatC(re_pop$tau2, digits = 3, format = "f")))))

dev.off()
cat("Population-based forest plot saved as 'forest_population_only.png'\n")
```

MACHINE LEARNING RANDOM FOREST MODEL PREDICTION OF VALIDATION SCORES

```
library(readxl)
library(randomForest)
library(caret)
library(ggplot2)
library(pdp)
library(vip)

data <- read_excel("EN.xlsx")
colnames(data) <- c("Study", "Sample_size", "Design", "Modality", "Score")

# Extract year
data$Year <- as.numeric(sub(".*?(\\d{4}).*", "\\1", data$Study))

# Convert categorical variables to factors
data$Design <- factor(data$Design)
data$Modality <- factor(data$Modality)
data$Score <- factor(data$Score, levels = c("1", "2", "3"), ordered = FALSE)

# Create binary outcome: Perfect score (3) vs Not perfect (1-2)
data$Perfect_score <- factor(ifelse(data$Score == 3, "Yes", "No"))

# Remove Study column for modeling (keep for reference)
data_model <- data[, c("Sample_size", "Design", "Modality", "Year", "Score",
"Perfect_score")]

cat("=== DATA SUMMARY ===\n")
print(summary(data_model))
cat("\nScore distribution:\n")
print(table(data_model$Score))
cat("\nPerfect score distribution:\n")
print(table(data_model$Perfect_score))
cat("\n")
```

APPROACH 1: PREDICT SCORE (1, 2, or 3) - MULTICLASS CLASSIFICATION

```
cat("=================================================================
=\n")
cat("APPROACH 1: PREDICTING VALIDATION SCORE (1, 2, or 3)\n")
cat("=================================================================
=\n\n")

# Set seed for reproducibility
set.seed(123)

# Split data: 70% training, 30% testing
train_indices <- createDataPartition(data_model$Score, p = 0.7, list = FALSE)
train_data <- data_model[train_indices, ]
test_data <- data_model[-train_indices, ]

cat("Training set size:", nrow(train_data), "\n")
cat("Test set size:", nrow(test_data), "\n\n")

cat("Training Random Forest model...\n")
rf_model_multiclass <- randomForest(
  Score ~ Sample_size + Design + Modality + Year,
  data = train_data,
  ntree = 500,             # Number of trees
  mtry = 2,                # Number of variables at each split
  importance = TRUE,       # Calculate variable importance
  proximity = TRUE,
  do.trace = 50            # Print progress every 50 trees
)

print(rf_model_multiclass)

cat("\n=== VARIABLE IMPORTANCE (MULTICLASS) ===\n")
importance_multiclass <- importance(rf_model_multiclass)
print(importance_multiclass)

png("rf_importance_multiclass.png", width = 10, height = 8, units = "in", res = 300)
varImpPlot(rf_model_multiclass,
        main = "Variable Importance for Predicting Validation Score (1-3)",
        pch = 19,
        col = "darkblue")
```

```
dev.off()
cat("\nVariable importance plot saved as 'rf_importance_multiclass.png'\n")

# Make predictions on the test set
predictions_multiclass <- predict(rf_model_multiclass, test_data)

# Confusion Matrix
cat("\n=== CONFUSION MATRIX (TEST SET) ===\n")
conf_matrix_multiclass <- confusionMatrix(predictions_multiclass, test_data$Score)
print(conf_matrix_multiclass)

# Model accuracy
cat("\nOverall Accuracy:", round(conf_matrix_multiclass$overall['Accuracy'], 3), "\n")
```

APPROACH 2: PREDICT PERFECT SCORE (YES/NO) - BINARY CLASSIFICATION

```
cat("\n===================================================================
===\n")
cat("APPROACH 2: PREDICTING PERFECT SCORE (YES vs NO)\n")
cat("===================================================================
=\n\n")

# Split data for binary classification
set.seed(123)
train_indices_binary <- createDataPartition(data_model$Perfect_score, p = 0.7, list = FALSE)
train_data_binary <- data_model[train_indices_binary, ]
test_data_binary <- data_model[-train_indices_binary, ]

# Train Random Forest for binary classification
cat("Training Random Forest model for binary outcome...\n")
rf_model_binary <- randomForest(
  Perfect_score ~ Sample_size + Design + Modality + Year,
  data = train_data_binary,
  ntree = 500,
  mtry = 2,
  importance = TRUE,
  proximity = TRUE,
  do.trace = 50
)

print(rf_model_binary)
```

```
# Variable Importance
cat("\n=== VARIABLE IMPORTANCE (BINARY) ===\n")
importance_binary <- importance(rf_model_binary)
print(importance_binary)

# Plot variable importance
png("rf_importance_binary.png", width = 10, height = 8, units = "in", res = 300)
varImpPlot(rf_model_binary,
        main = "Variable Importance for Predicting Perfect Score",
        pch = 19,
        col = "darkred")
dev.off()
cat("\nVariable importance plot saved as 'rf_importance_binary.png'\n")

# Make predictions
predictions_binary <- predict(rf_model_binary, test_data_binary)

# Get prediction probabilities
predictions_prob <- predict(rf_model_binary, test_data_binary, type = "prob")

# Confusion Matrix
cat("\n=== CONFUSION MATRIX (BINARY - TEST SET) ===\n")
conf_matrix_binary <- confusionMatrix(predictions_binary,
test_data_binary$Perfect_score)
print(conf_matrix_binary)

# Model performance metrics
cat("\n=== MODEL PERFORMANCE METRICS ===\n")
cat("Accuracy:", round(conf_matrix_binary$overall['Accuracy'], 3), "\n")
cat("Sensitivity:", round(conf_matrix_binary$byClass['Sensitivity'], 3), "\n")
cat("Specificity:", round(conf_matrix_binary$byClass['Specificity'], 3), "\n")
cat("Precision:", round(conf_matrix_binary$byClass['Pos Pred Value'], 3), "\n")
cat("F1 Score:", round(conf_matrix_binary$byClass['F1'], 3), "\n")
```

VARIABLE IMPORTANCE ANALYSIS

```
cat("\n=================================================================
===\n")
cat("DETAILED VARIABLE IMPORTANCE ANALYSIS\n")
```

```
cat("==========================================================================
=\n\n")

# Extract and rank importance
importance_df <- data.frame(
  Variable = rownames(importance_binary),
  MeanDecreaseAccuracy = importance_binary[, "MeanDecreaseAccuracy"],
  MeanDecreaseGini = importance_binary[, "MeanDecreaseGini"]
)
importance_df <- importance_df[order(-importance_df$MeanDecreaseAccuracy), ]

cat("Variables ranked by importance (MeanDecreaseAccuracy):\n")
print(importance_df)

# Enhanced importance plot using vip package
png("rf_importance_enhanced.png", width = 10, height = 8, units = "in", res = 300)
vip(rf_model_binary,
    num_features = 4,
    bar = TRUE,
    horizontal = TRUE,
    aesthetics = list(fill = "steelblue", color = "black")) +
  ggtitle("Variable Importance for Predicting Perfect Validation Score") +
  theme_minimal(base_size = 14)
dev.off()
cat("\nEnhanced importance plot saved as 'rf_importance_enhanced.png'\n")
```

ROC CURVE ANALYSIS

```
cat("==========================================================================
=\n")
cat("ROC CURVE ANALYSIS\n")
cat("==========================================================================
=\n\n")

library(pROC)

# Get predicted probabilities for the positive class (Perfect score = "Yes")
# Make sure to get probabilities for test set
pred_prob_test <- predict(rf_model_binary, test_data_binary, type = "prob")

# Create ROC object
```

```r
roc_obj <- roc(test_data_binary$Perfect_score, pred_prob_test[, "Yes"])

# Print ROC statistics
cat("=== ROC CURVE STATISTICS ===\n")
cat("AUC (Area Under Curve):", round(auc(roc_obj), 4), "\n")
cat("95% CI for AUC:",
    paste(round(ci.auc(roc_obj)[c(1,3)], 4), collapse = " - "), "\n\n")

# Find optimal threshold using Youden's Index
coords_obj <- coords(roc_obj, "best", best.method = "youden")
cat("Optimal Threshold (Youden's Index):", round(coords_obj$threshold, 4), "\n")
cat("  Sensitivity:", round(coords_obj$sensitivity, 4), "\n")
cat("  Specificity:", round(coords_obj$specificity, 4), "\n\n")


# Enhanced ROC plot with ggplot2
library(ggplot2)

# Extract ROC data
roc_data <- data.frame(
  sensitivity = roc_obj$sensitivities,
  specificity = roc_obj$specificities,
  threshold = roc_obj$thresholds
)

# Calculate 1 - specificity for x-axis
roc_data$fpr <- 1 - roc_data$specificity

# Create enhanced plot
png("roc_curve_enhanced.png", width = 10, height = 10, units = "in", res = 300)
p <- ggplot(roc_data, aes(x = fpr, y = sensitivity)) +
  geom_line(color = "darkblue", size = 1.5) +
  geom_abline(slope = 1, intercept = 0, linetype = "dashed",
              color = "gray50", size = 1) +
  geom_point(data = data.frame(
    fpr = 1 - coords_obj$specificity,
    sensitivity = coords_obj$sensitivity
  ), aes(x = fpr, y = sensitivity),
  color = "red", size = 4, shape = 19) +
  annotate("text",
           x = 0.6, y = 0.2,
           label = paste0("AUC = ", round(auc(roc_obj), 3)),
```

```
         size = 6, fontface = "bold") +
  annotate("text",
         x = 1 - coords_obj$specificity + 0.05,
         y = coords_obj$sensitivity + 0.05,
         label = paste0("Optimal threshold\n(",
                  round(coords_obj$threshold, 3), ")"),
         size = 4, color = "red") +
  labs(
    title = "ROC Curve: Random Forest Prediction of Perfect Validation Score",
    subtitle = paste0("AUC = ", round(auc(roc_obj), 4),
                " [95% CI: ",
                paste(round(ci.auc(roc_obj)[c(1,3)], 3), collapse = "-"),
                "]"),
    x = "False Positive Rate (1 - Specificity)",
    y = "True Positive Rate (Sensitivity)"
  ) +
  theme_minimal(base_size = 14) +
  theme(
    plot.title = element_text(hjust = 0.5, face = "bold", size = 16),
    plot.subtitle = element_text(hjust = 0.5, size = 12),
    panel.grid.minor = element_blank(),
    panel.border = element_rect(color = "black", fill = NA, size = 1)
  ) +
  coord_fixed(ratio = 1) +
  scale_x_continuous(limits = c(0, 1), breaks = seq(0, 1, 0.2)) +
  scale_y_continuous(limits = c(0, 1), breaks = seq(0, 1, 0.2))

print(p)
dev.off()
cat("Enhanced ROC curve saved as 'roc_curve_enhanced.png'\n")
```

Compare with the training set ROC (to check overfitting)

```
pred_prob_train <- predict(rf_model_binary, train_data_binary, type = "prob")
roc_train <- roc(train_data_binary$Perfect_score, pred_prob_train[, "Yes"])

cat("\n=== TRAIN vs TEST COMPARISON ===\n")
cat("Training AUC:", round(auc(roc_train), 4), "\n")
cat("Test AUC:", round(auc(roc_obj), 4), "\n")
cat("Difference:", round(auc(roc_train) - auc(roc_obj), 4), "\n")
if (auc(roc_train) - auc(roc_obj) > 0.1) {
```

```
  cat("WARNING: Large difference suggests possible overfitting!\n")
} else {
  cat("Model generalizes well (no major overfitting detected).\n")
}
cat("\n")


# Plot training vs test ROC curves
png("roc_train_vs_test.png", width = 10, height = 10, units = "in", res = 300)
plot(roc_train,
     main = "ROC Curves: Training vs Test Set",
     col = "darkorange",
     lwd = 2,
     legacy.axes = TRUE,
     grid = TRUE)
plot(roc_obj, add = TRUE, col = "purple", lwd = 2)
abline(a = 0, b = 1, lty = 2, col = "gray50")
legend("bottomright",
       legend = c(paste0("Training (AUC = ", round(auc(roc_train), 3), ")"),
                  paste0("Test (AUC = ", round(auc(roc_obj), 3), ")"),
                  "Reference"),
       col = c("darkorange", "purple", "gray50"),
       lwd = c(2, 2, 1),
       lty = c(1, 1, 2),
       bty = "n",
       cex = 1.2)
dev.off()
cat("Train vs Test ROC curve saved as 'roc_train_vs_test.png'\n\n")
```
