# Supplementary material for: Endophenotype Research in Epilepsy Across Time
Source: Brain Sci. 2025 Nov 27;15(12):1275. doi: 10.3390/brainsci15121275 (PMC12730710; doi:10.3390/brainsci15121275)
Supplement: Supplementary file 1 [file brainsci-15-01275-s001.zip › Supplementary Table S4-NOS.pdf]

	Author, Year, Theme, Reference Number	Study Design Type	NOS type	NOS Score (Reviewer 1)	NOS Score (Reviewer 2)	Risk of Bias
1	Sancetta et al. (2025) Altered neural avalanche spreading in DRE [56]	Retrospective cohort design with comparison groups	NOS for Cohort Studies	9/9	9/9	Low
2	Struck et al. (2025) JME Imaging Endophenotypes [45]	Cross-sectional cohort study design with comparison groups	NOS for Cohort Studies	8/9	8/9	Low
3	Rossi et al. (2025) GABRG2 Loss- and Gain-of-Function Variants [78]	Cross-Sectional Cohort Study with Functional Analysis	NOS for Cohort Studies	8/9	8/9	Low
4	Gavnholt et al. (2025) Clustering IGE patients' phenotypes [72]	Cross-Sectional Cohort Study	NOS for Cohort Studies (Cross-Sectional)	8/9	8/9	Low
5	Gesche et al. (2024) Prodromal Phase of IGE [86]	Case-Control study	NOS for Case-Control Studies	9/9	9/9	Minimal
6	Asha et al. (2024) EEG microstate parameters in different epilepsy types [44]	Cross-Sectional Cohort Study with Comparison Groups	NOS for Cohort Studies (Cross-Sectional)	8/9	8/9	Low
7	Badura-Stronka et al. (2024) Polish epilepsy genetic study [87]	Cross-sectional genetic cohort design	NOS for Cohort Studies	8/9	8/9	Low
8	Casella et al. (2024) Cortical microstructure alterations in pediatric focal epilepsy [54]	Cross-Sectional Cohort Study with Comparison Groups	NOS for Cohort Studies	8/9	8/9	Low

9	Caciagli et al. (2023) Cognitive phenotype in JAE and their siblings [66]	Prospective Cross-Sectional Cohort Study with Family Component	NOS for Cohort Studies (Cross-Sectional)	8/9	8/9	Low
10	Hershberger et al. (2023) Molecular subtypes and post-surgical seizure recurrence [81]	Prospective Cohort Study with Outcome Follow-up	NOS for Cohort Studies	9/9	9/9	Minimal
11	Jeppesen et al. (2023) Neuropsychological phenotype in IGE [73]	Cross-Sectional Cohort	NOS for Cohort Studies (Cross-Sectional)	8/9	8/9	Low
12	Wang et al. (2023) Functional network abnormalities in TLE and their siblings [51]	Cross-sectional cohort study with unaffected sibling comparison	NOS for Cohort Studies (Cross-Sectional)	8/9	8/9	Low
13	Stier et al. (2022) EEG/MEG synchrony, cortical thinning in GGE and their siblings [62]	Cross-sectional cohort study with unaffected sibling comparison	NOS for Cohort Studies (Cross-Sectional)	8/9	8/9	Low
14	Maes et al. (2022) Comorbid psychiatric disorders in TLE [38]	Cross-sectional case-control study	NOS for Case-Control Studies	7/9	8/9	Low-Moderate
15	Irelli et al. (2022) Long-term seizure outcomes in EEM [83]	Retrospective multicenter cohort study with prospective follow-up	NOS for Cohort Studies	8/9	8/9	Low
16	Ur-Özçelik et al. (2021) JME Photosensitivity [40]	Cross-sectional cohort study with photosensitivity subgroup analysis	NOS for Cohort Studies (Cross-Sectional)	8/9	8/9	Low

17	Stier et al. (2021) MEG Network Heritability in GGE and their siblings [41]	Cross-sectional cohort study with unaffected sibling comparison	NOS for Cohort Studies (Cross-Sectional)	8/9	8/9	Low
18	Gesche et al. (2021) MEP Polyphasia Study in IGE [59]	Cross-sectional cohort study	NOS for Cohort Studies (Cross-Sectional)	7/9	7/9	Low-Moderate
19	Clemens et al. (2021) Resting-state EEG theta activity study [60]	Cross-sectional cohort study	NOS for Cohort Studies (Cross-Sectional)	7/9	7/9	Low-Moderate
20	Zhu et al. (2020) SCN8A Variant Family Neuroimaging Study [88]	Cross-sectional family-based cohort study with unaffected sibling comparison	NOS for Cohort Studies (Cross-Sectional)	8/9	8/9	Low
21	Yaakub et al. (2020) mTLE Alpha Network Endophenotypes and relatives [63]	Cross-sectional cohort study with unaffected sibling comparison	NOS for Cohort Studies (Cross-Sectional)	8/9	8/9	Low
22	Tan et al. (2020) Cognitive Impairments in TLE pts and their siblings [67]	Cross-sectional cohort study with unaffected sibling comparison	NOS for Cohort Studies (Cross-Sectional)	8/9	8/9	Low
23	de Lange et al. (2020) Modifier genes in *SCN1A*-related epilepsy [77]	Cross-sectional genetic cohort study	NOS for Cohort Studies (Cross-Sectional)	8/9	8/9	Low
24	Caciagli et al. (2020) Motor hyperactivation in fMRI in JME patients and their siblings	Cross-sectional cohort study with unaffected sibling comparison	NOS for Cohort Studies (Cross-Sectional)	8/9	8/9	Low

	[46]					
25	Long et al. (2020) Hippocampal abnormalities in sporadic TLE and their siblings [50]	Cross-sectional cohort study with unaffected sibling comparison	NOS for Cohort Studies (Cross-Sectional)	8/9	8/9	Low
26	Gesche (2020) The clinical spectrum of familial and sporadic IGE [85]	Cross-sectional cohort study (NOT case-control)	NOS for Cohort Studies (Cross-Sectional)	7/9	7/9	Low-Moderate
27	Caciagli et al. (2019) Abnormal hippocampal structure and function in JME and their siblings [48]	Cross-sectional cohort study with unaffected sibling comparison	NOS for Cohort Studies (Cross-Sectional)	8/9	8/9	Low
28	Yaakub et al. (2019) Temporal Lobe Morphology in mTLE+HS and relatives [53]	Cross-sectional cohort study with unaffected sibling comparison	NOS for Cohort Studies (Cross-Sectional)	8/9	8/9	Low
29	Wandschneider et al. (2019) Developmental MRI Markers in JME and Siblings [49]	Cross-sectional cohort study with unaffected sibling comparison	NOS for Cohort Studies (Cross-Sectional)	8/9	8/9	Low
30	Tangwiriyasakul et al. (2019) Functional hypersynchrony in sensorimotor fMRI in GGE and relatives [42]	Cross-sectional cohort study with unaffected first-degree relative comparison	NOS for Cohort Studies (Cross-Sectional)	8/9	8/9	Low
31	Schraegle (2017) Paternal anxiety in pediatric epilepsy and their fathers [39]	Cross-sectional cohort study	NOS for Cohort Studies (Cross-Sectional)	7/9	8/9	Low-Moderate
32	Wight et al. (2016)	Family-based genetic linkage study	NOS for Cohort Studies	8/9	8/9	Low

	Genetic Linkage in JME Subsyndromes [79]		(Cross-Sectional)			
33	Carvalho et al. (2016) Cognitive performance in JME [71]	Cross-sectional cohort study	NOS for Cohort Studies (Cross-Sectional)	7/9	7/9	Low-Moderate
34	Shen et al. (2015) BDNF Val66Met in TLE [80]	Case-control genetic association study	NOS for Case-Control Studies	7/9	7/9	Low-Moderate
35	Chowdhury et al. (2015) Motor evoked potential polyphasia in IGE and first-degree relatives [64]	Cross-sectional cohort study with unaffected first-degree relative comparison	NOS for Cohort Studies (Cross-Sectional)	8/9	8/9	Low
36	Iqbal et al., (2015) Neurocognitive in JME and their siblings [70]	Cross-sectional cohort study with unaffected sibling comparison	NOS for Cohort Studies (Cross-Sectional)	8/9	8/9	Low
37	Uchida et al. 2015 Praxis Induction in JME [74]	Prospective cohort study with prognostic phenotype analysis	NOS for Cohort Studies	8/9	8/9	Low
38	Whelan et al. (2015) White matter alterations in TLE and their siblings [55]	Cross-sectional cohort study with unaffected sibling comparison	NOS for Cohort Studies (Cross-Sectional)	8/9	8/9	Low
39	Addis et al. (2014) Migraine in Rolandic Epilepsy [84]	Family-based genetic linkage study	NOS for Cohort Studies (Cross-Sectional)	8/9	8/9	Low
40	Chowdhury et al. (2014) Cognitive Endophenotypes in IGE and their relatives [68]	Cross-sectional cohort study with unaffected first-degree relative comparison	NOS for Cohort Studies (Cross-Sectional)	8/9	8/9	Low

41	Wandschneider et al. (2014) Motor co-activation in JME and their siblings [47]	Cross-sectional cohort study with unaffected sibling comparison	NOS for Cohort Studies (Cross-Sectional)	8/9	8/9	Low
42	Chowdhury et al. (2014) Brain network study in IGE and its relatives [61]	Cross-sectional cohort study with unaffected first-degree relative comparison	NOS for Cohort Studies	8/9	8/9	Low
43	Alhusaini et al. (2013) Subcortical Volume Heritability in MTLE and their siblings [43]	Cross-sectional cohort study with unaffected sibling comparison	NOS for Cohort Studies (Cross-Sectional)	8/9	8/9	Low
44	Tikka et al. (2013) Quantitative EEG in JME [37]	Cross-sectional cohort study	NOS for Cohort Studies (Cross-Sectional)	6/9	7/9	Moderate
45	Scanlon et al., (2013) Brain Structure Volumes in TLE and their siblings [52]	Cross-sectional cohort study with unaffected sibling comparison	NOS for Cohort Studies (Cross-Sectional)	8/9	8/9	Low
46	Verotti et al. (2013) Neuropsychological impairment in Rolandic Epilepsy pts and their siblings [69]	Cross-sectional cohort study with unaffected sibling comparison	NOS for Cohort Studies (Cross-Sectional)	7/9	7/9	Low-Moderate
47	Smith et al. (2012) Neurocognitive endophenotype in Rolandic epilepsy patients and their siblings [89]	Cross-sectional cohort study with unaffected sibling comparison	NOS for Cohort Studies (Cross-Sectional)	7/9	7/9	Low-Moderate
48	Clemens et al. (2012) EEG-LORETA Endophenotypes of IGE	Cross-sectional cohort study	NOS for Cohort Studies (Cross-Sectional)	7/9	7/9	Low-Moderate

	[65]					
49	Beniczky et al. (2012) Reflex epileptic traits in JME [75]	Cross-sectional cohort study	NOS for Cohort Studies (Cross-Sectional)	7/9	7/9	Low-Moderate
50	Guaranha et al. (2011) Reflex/psychiatric traits in JME prognosis [76]	Prospective cohort study with prognostic analysis	NOS for Cohort Studies	8/9	8/9	Low
51	Brazzo et al. (2011) EEG and pattern reversal visual evoked potential assessment in photosensitive epilepsy and IGE [57]	Cross-sectional cohort study	NOS for Cohort Studies (Cross-Sectional)	8/9	8/9	Low
52	Mula et al. (2010) – Interictal Dysphoric Disorder [82]	Cross-sectional cohort study	NOS for Cohort Studies (Cross-Sectional)	7/9	7/9	Low-Moderate
53	Boutros et al., 2006 Auditory-Evoked Responses and Sensory Gating in Focal Epilepsy [58]	Cross-sectional cohort study	NOS for Cohort Studies	7/9	7/9	Low-Moderate
