# Supplementary material for: Endophenotype Research in Epilepsy Across Time
Source: Brain Sci. 2025 Nov 27;15(12):1275. doi: 10.3390/brainsci15121275 (PMC12730710; doi:10.3390/brainsci15121275)
Supplement: Supplementary file 1 [file brainsci-15-01275-s001.zip › Supplementary Table S5-GRADE.pdf]

Supplementary Table S5: GRADE-Style Certainty of Evidence Assessment by Endophenotype Class

Endophenotype Class	No. Studies	Certainty Level (GRADE)	Validation Rate	Key Strengths	Key Limitations	Clinical Readiness
Neuroimaging	18	HIGH	77.8%	Strong family study data; robust, consistent findings	Mostly European ancestry; equipment cost	Moderate-High
Electro-physiological	11	MODERATE	27.3%	Non-invasive; rapid; cost-effective	Poor standardization; high heterogeneity	Low-Moderate
Cognitive	10	MODERATE	60%	Accessible; validated tests; replicable	Variability in batteries; confounders	Moderate-High
Genetic/Molecular	6	MODERATE-HIGH	83.3%	Direct genetic data; clinical testing available	Limited non-European data; complex genetics	Moderate-High
Psychiatric	3	MODERATE	Variable	Non-invasive; rapid; cost-effective	Limited studies; no standard protocol	Low
Clinical Phenotyping	5	MODERATE	Variable	Non-invasive; rapid; cost-effective	Limited studies; no standard protocol	Low
